# Hearing loss genes reveal patterns of adaptive evolution at the coding and non-coding levels in mammals

**DOI:** 10.1186/s12915-021-01170-6

**Published:** 2021-11-16

**Authors:** Anabella P. Trigila, Francisco Pisciottano, Lucía F. Franchini

**Affiliations:** 1 Instituto de Investigaciones en Ingeniería Genética y Biología Molecular (INGEBI), Consejo Nacional de Investigaciones Científicas y Técnicas (CONICET), C1428, Buenos Aires, Argentina; 2 Current address: Instituto de Biología y Medicina Experimental (IBYME), Consejo Nacional de Investigaciones Científicas y Técnicas (CONICET), C1428, Buenos Aires, Argentina

**Keywords:** Inner ear, Evolution, Hearing loss, Mammals, HARs, TSARs

## Abstract

**Background:**

Mammals possess unique hearing capacities that differ significantly from those of the rest of the amniotes. In order to gain insights into the evolution of the mammalian inner ear, we aim to identify the set of genetic changes and the evolutionary forces that underlie this process. We hypothesize that genes that impair hearing when mutated in humans or in mice (hearing loss (HL) genes) must play important roles in the development and physiology of the inner ear and may have been targets of selective forces across the evolution of mammals. Additionally, we investigated if these HL genes underwent a human-specific evolutionary process that could underlie the evolution of phenotypic traits that characterize human hearing.

**Results:**

We compiled a dataset of HL genes including non-syndromic deafness genes identified by genetic screenings in humans and mice. We found that many genes including those required for the normal function of the inner ear such as *LOXHD1*, *TMC1*, *OTOF*, *CDH23*, and *PCDH15* show strong signatures of positive selection. We also found numerous noncoding accelerated regions in HL genes, and among them, we identified active transcriptional enhancers through functional enhancer assays in transgenic zebrafish.

**Conclusions:**

Our results indicate that the key inner ear genes and regulatory regions underwent adaptive evolution in the basal branch of mammals and along the human-specific branch, suggesting that they could have played an important role in the functional remodeling of the cochlea. Altogether, our data suggest that morphological and functional evolution could be attained through molecular changes affecting both coding and noncoding regulatory regions.

**Supplementary Information:**

The online version contains supplementary material available at 10.1186/s12915-021-01170-6.

## Background

Mammals possess unique hearing capacities that differ significantly from those of the rest of the amniotes. They enjoy the widest hearing ranges of the animal world and a great diversity in tuning that allow them to detect the most extreme high and low hearing frequencies. This is facilitated by its unique cochlear anatomy, which is the morphological base of high-frequency responsiveness. The organ of Corti, elongated and coiled in therian mammals, favored an improved auditory transmission by hosting several rows of hair cells, the specialized hearing sensing cells of this system [1–3].

It has been proposed that the evolutionary changes that rendered the mammalian cochlea and its unique characteristics were deeply guided by adaptive evolution, recognizable at the molecular level by strong signatures of positive selection in the mammalian lineage for a group of particular inner ear genes [4–7].

In this work, we aimed to perform a comprehensive identification of genetic modifications and evolutionary mechanisms that underlie the evolution of the functional and morphological novelties of the mammalian inner ear. We hypothesize that this organ’s uniqueness emerged as the result of protein-coding as well as noncoding regions’ adaptive changes in the mammalian basal branch. However, the relative contribution of each one of these functional compartments into the evolution of the mammalian inner ear had not been previously studied. “Which is the locus for evolution?” is a hot topic of debate within the evolutionary community that tries to address whether genetic changes underlying morphological evolution occur in coding or non-coding regions of the genome [8–10]. Motivated by this open discussion, we set out to explore this broad question by studying the evolutionary mechanisms underlying morphological and functional evolution in the mammalian inner ear. However, to identify the functional genetic elements that were remodeled and the evolutionary forces underlying the evolution of the mammalian ear is not an easy task. First, the inner ear transcriptome is composed of many genes that are also expressed in other tissues and organs, with a few genes having exclusively inner ear known expression [11]. Second, according to the classical evo-devo paradigm [10, 12], gene developmental programs have to be modified to have an impact on morphological and functional evolution. However, since developmental genes are very sensitive to mutations and changes, they are in general under strong purifying selection and it has been proposed that potential modifications of these genes occur through regulatory changes [9, 12]. To approach these issues, we first decided to compile a dataset of genes that are well documented as being involved in hearing function (hearing loss genes; HL genes), and then, we performed a comprehensive positive selection analysis in coding regions and explored if cis-regulatory elements related to HL genes were targets of non-coding accelerated evolution in the mammalian lineage.

Additionally, we investigated if these HL genes underwent a human-specific evolutionary process that could underlie the predisposition to hearing loss and the evolution of phenotypic traits that characterize human hearing. Unlike the general mammalian trend, the hearing range of humans is restricted to relatively low frequencies. In the lineage leading to humans and chimpanzees, it has been shown that cochlear size increased significantly, probably enabling better low-frequency hearing [13]. This trend could be explained simply as a result of increasing animal size or as driven by positive selection to improve low-frequency communication abilities. Since hearing is the main source for communication in humans and its related disorders may impair the ability to use speech, it has been even speculated that, only in the human lineage, the processing of speech signals has acted as a selective force on cochlear evolution [14]. This could be a result of human speech mainly being composed of lower frequencies since humans have one of the lowest upper-frequency limits of all mammals and display high-frequency selectivity at low frequencies [13, 15]. Oral language understanding is a key component for the development of several cognitive tasks and humans are the only primate species with a controlled spoken production that can be socially learned, becoming a part of traditionally shared communication [16]. In order to examine if key inner ear genes have evolved in a human-specific manner, we searched for evidence of coding and noncoding signatures of positive selection in the human lineage in hearing loss genes. The evolution of hearing genes in humans has not been explored before and it could shed light on the emergence of functional properties of human hearing that could be related to the parallel evolution of hearing and speech.

## Results

### Hearing loss gene dataset

**Fig. 1.**
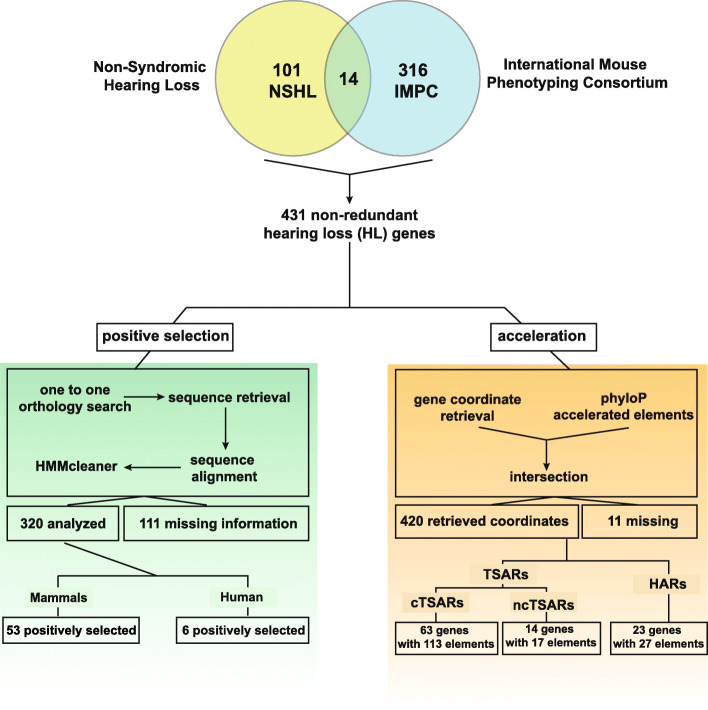
Bioinformatics pipeline and filtering process of two datasets of hearing loss genes: the classic dataset composed of non-syndromic hearing loss genes identified in humans which is composed of a total of 129 coding genes obtained by combining the information available in the “Hereditary Hearing Loss Homepage” [17], the “Genetics Home Reference – Nonsyndromic deafness related genes” website [18], the Connexin-deafness homepage – Nonsyndromic deafness related connexins” [19] and from the chapter “Deafness and Hereditary Hearing Loss Overview” from the online book “Gene Review” [20], and the recently identified hearing impairment genes by the International Mouse Phenotype Consortium [21–23]

With the aim of testing the prevalence of Darwinian selection in a set of well-known inner ear genes, we built a local database of HL genes including reported genes that had been linked to deafness and hearing loss from various sources. First, we combined available information from the “Hereditary Hearing Loss Homepage” [17], the “Genetics Home Reference – Nonsyndromic deafness related genes” website [18], the Connexin-deafness homepage “Nonsyndromic deafness related connexins” [19] and from the chapter “Deafness and Hereditary Hearing Loss Overview” from the online book “Gene Review” [20]. This combination of non-syndromic hearing loss (NSHL) databases allowed for the initial identification of 115 disease-causing genes (Fig. 1). We also included recently identified novel candidate hearing loss genes detected by the International Mouse Phenotyping Consortium (IMPC [21–23];). This organization identified 330 candidate hearing loss genes through the diagnosis of aberrant ABRs from a total set of 3006 mutant mice explored (Fig. 1). As a result, our non-redundant HL genes database (HLGD) was composed of 431 genes, with 14 genes being common to the NSHL and IMPC datasets (Additional file 1: Table S1; Fig. 1). Coding and noncoding regions of these HL gene transcriptional units were subjected to evolutionary analysis.

### High-throughput screening of mammalian positive selection at the coding level

As an initial approach to inspect for signatures of positive selection in the mammalian basal branch, we were able to retrieve complete coding sequence information for fourteen species for 320 genes of our HLGD and we analyzed them through an adaptive evolutionary analysis (Fig. 1). We were not able to analyze 111 HL genes because 100 presented missing sequence orthologs in more than one species and/or displayed one-to-many or many-to-many orthology relationships involving one or more species and 11 had no information in our reference genome (Additional file 1: Table S1; Fig. 1). We used the positive selection modified Model A branch-site test 2 [24] implemented through the *codeml* program from the PAML4 package [25] using two different alignment methods. The intersection of these positive selection tests revealed that 58 candidate genes out of the 320 analyzed have a signature (*p* < 0.05) of adaptive evolution at the basal mammalian lineage (Supp. Table S1). This initial set of candidate genes were subjected to a more deep scrutiny of their coding sequences (see next section). Additionally, we complemented our Darwinian selection examination with information from available accelerated regions in therian mammals [26]. In order to identify non-coding accelerated evolution in HL genes, we used a database of conserved regions showing signatures of therian mammal-specific acceleration [TSARs [26];]. Since TSAR elements include coding and non-coding regions, we classified this database in coding TSARs (cTSARs) containing regions that overlapped exonic elements and noncoding TSARs (ncTSARs). We used the dataset of cTSARs to perform a search over 420 genomic coordinates including the coding regions of our non-redundant database of HL genes. Eleven genes were unique to the mouse genome and did not have an ortholog in humans, and therefore, coordinate retrieval was not possible (Additional file 1: Table S1). This approach allowed us to find signatures of accelerated evolution at the coding level for genes that either did not display signatures of positive selection through the PAML approach or could not be analyzed using this method due to missing sequence information or multiple orthology. We established that 63 genes had 113 cTSARs spanning at least one coding exon (Fig. 1; Supp. Table S1). Notably, among the genes that resulted negative in the PAML analysis for positive selection, we found several genes that showed a high number of TSARs in its coding portion: for instance, the gene *GPR156* contained 8 TSAR elements (Supp. Table S1). Regarding genes that were not analyzed through PAML due to missing sequence information, we detected 7 genes (*COL11A2*, *FBXL8*, *FGFR4*, *LDHB*, *MYH1*, *MYO15A*, *RYR1*) that showed accelerated regions in its coding sequence. Among them, it is noticeable some genes displaying several cTSARs, such as *FGFR4* and *RYR1* (Additional file 1: Table S1). The presence of several cTSARs could indicate that several coding portions of these genes underwent extensive sequence remodeling in the mammalian lineage, particularly in the therian branch.

### Extended analysis of mammalian positively selected genes

Hearing loss genes detected as positive selected in the high-throughput analysis (*codeml* codonFreq =3 FDR < 0.05) were analyzed in more detail expanding the number of species used, thus increasing the statistical power to detect specific codon sites that display signatures of Darwinian selection. We performed an individual analysis of each of these positively selected genes by increasing the number of species of the high throughput analysis. As a result, a total of 58 genes were re-analyzed with at least 38 species and positive selection was confirmed for 53 of them including *SLC26A5*, the gene encoding prestin, which has been previously reported [4] (Table 1; Additional file 1: Table S2). Of our reported final set, 48 genes were not previously reported as positively selected in the mammalian basal lineage and only 5 (Additional file 1: Table S2) were formerly identified in a study exploring selection in the inner ear transcriptome [6].

**Table 1:** The 53 coding positively selected hearing loss genes in the mammalian basal branch

Positively selected hearing loss genes in the mammalian basal branch	
*ADGRB1, AFF3, ANKRD11, ARHGAP23, ASPM, ATP2B2, ATP2B4, CASZ1, CCDC127, CDH23, COL9A2, CRYM, DBN1, DCDC2, DIAPH3, DMXL2, ELMO3, ESPN, ESRRB, F13A1, FMNL3, GAS2L1, GAS6, GSTCD, GTPBP2, KCNQ4, LOR, LOXHD1, MOGS, MYH14, MYO1A, MYO3A, NHSL1, NIN, NISCH, OTOF, PCDH15, PHF3, PLCB2, RASD1, RNF10, SCARF2, SCN4A, SCRIB, SERINC3, SH3TC2, SLC26A5, SRRM4, TMC1, TMEM132E, TNC, VWA3A, WHRN*	

**Fig. 2.**
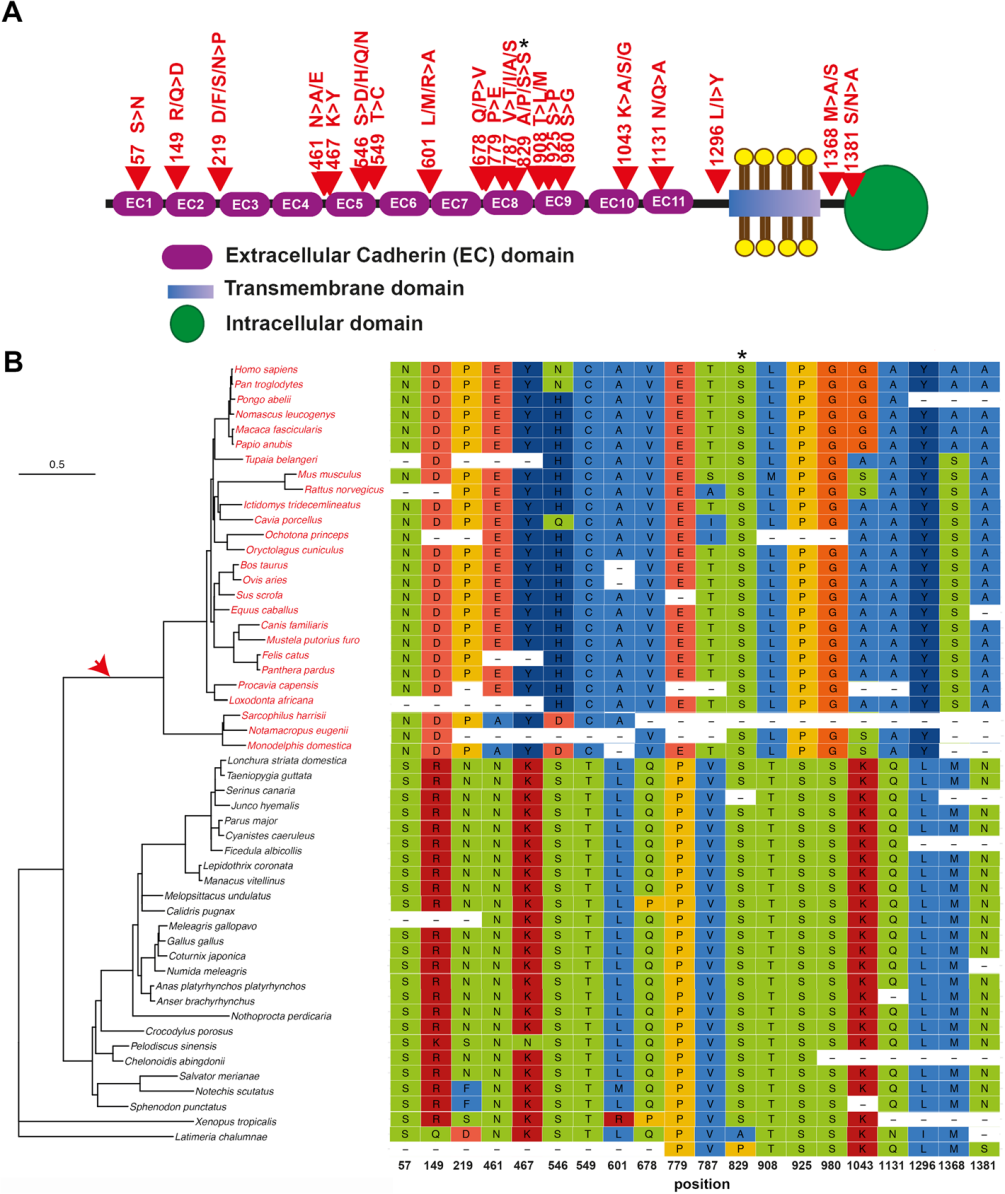
Phylogenetic tree and positive selected sites of an essential tip link protein: PCDH15. **A** Schematic diagram of the PCDH15 protein domains showing the approximate localization of positively selected sites (red arrows). Protein domains were approximately depicted according to UniProtKB - A9Z1W1 (A9Z1W1_HUMAN). **B** On the left: phylogenetic tree showing vertebrate species used for the analysis. The red arrow indicates the foreground branch. Mammalian species included in the foreground branch are in red. On the right: sequence alignment of positively selected sites identified. Positions of the human sequence given correspond to the ENST00000373965.6 transcript. Note that a change of Serine by Serine (asterisk) is identified as a positive selected site: serine is the only amino acid that is encoded by two disjoint codon sets so that a tandem substitution of two nucleotides is required to switch between the two sets. It has been suggested that the great majority of codon set switches proceed by two consecutive nucleotide substitutions, via a threonine or cysteine intermediates, are driven by selection even though this may not be the kind of positive selection driving functional divergences [29, 30]

**Fig. 3.**
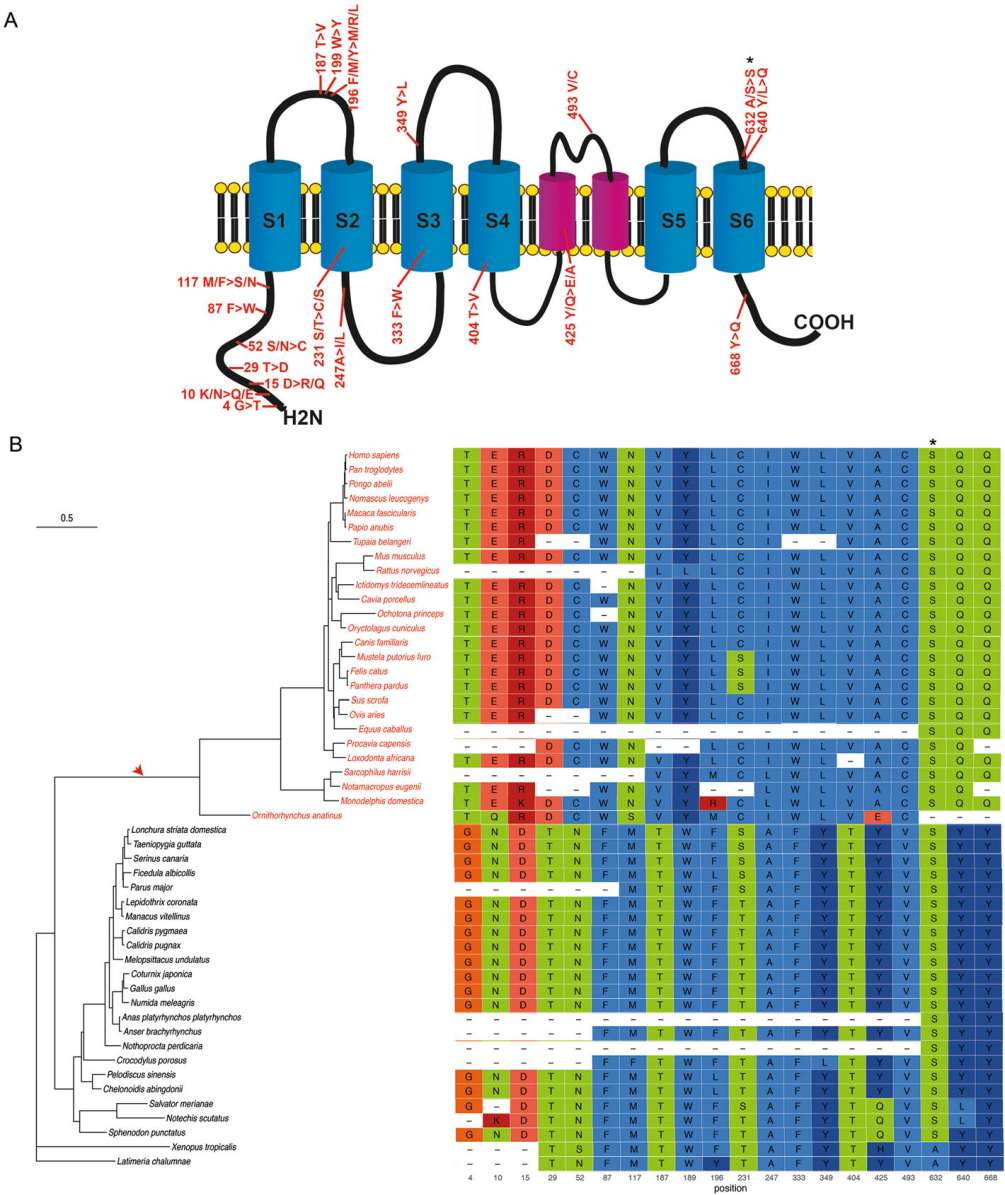
Phylogenetic tree and positive selected sites of an essential tip link protein: TMC1. **A** Schematic diagram of the TMC1 protein domains (taken from [40]) showing the approximate localization of positively selected sites. Transmembrane domains (S1 to S6) were depicted using info from UniProtKB - Q8TDI8 (TMC1_HUMAN). **B** On the left: phylogenetic tree showing vertebrate species used for the analysis. The red arrow indicates the foreground branch. Mammalian species included in the foreground branch are written in red. On the right: sequence alignment of the positively selected sites identified. Positions of the human sequence given correspond to the ENST00000645208.2 transcript. Note that a change of Serine by Serine (asterisk) is identified as a positive selected site, see Fig. 2 legend for an explanation

Thus, we were able to confirm that most genes identified in our first screening (53 out of 58) underwent adaptive evolution in the mammalian lineage and we could also identify numerous codon sites showing signatures of positive selection (positive selected sites: PSSs. BEB > 0.95; Additional file 1: Table S2). Among these genes are noteworthy *Protocadherin Related 15* (*PCDH15*), *Transmembrane Channel Like 1* (*TMC1*), and *Cadherin Related 23* (*CDH23*) which have been largely regarded as involved in the mechanotransduction of the inner ear [27, 28] and display many sites with signatures of Darwinian selection (Additional file 1: Table S2). In fact, *PCDH15* and *CDH23*, members of the tip link, display many positively selected sites located at key functional regions such as the cadherin domains (EC): EC1, EC2, EC5, EC8, EC9, EC10, and EC11 in PCDH15 (Fig. 2A, B; Additional file 1: Table S2) and EC10, EC11, EC15, and EC23 in CDH23 (Additional file 2: Fig. S 1[31];). Interestingly, the PSSs in PCDH15 are located in both the initial cadherin domains that interact with CDH23 and also in those near the C-terminal: EC8, EC9, EC10, and EC11. The arrangement of these latter domains is unusual, and they have been suggested to alter the elastic response of the tip link [32]. The PSSs in the bent calcium-free region EC9-EC10 (Fig. 2A) might be relevant for the mechanical function in sensory perception and hair-cell transduction. After the EC11, PCDH15 has a conserved region of an unknown structure named as Protocadherin 15 Interacting-Channel Associated (PICA) which was suggested to be the site of ion-channel interaction (Fig. 2A). The PICA domain has been identified to mediate cis-dimerization of PCDH15 [33] and holds two positively selected sites in this region, close to the beginning of the transmembrane domain (Fig. 2A, B). These sites, along with another one (found in the region close to EC11), are located in PCDH15 protein domains that have been suggested to interact with TMC1/TMC2, TMIE, and TMHS [[34–36] reviewed in [37]]. One of these genes, *TMC1*, which has been postulated to code for the mechanotransduction channel [38, 39], displays numerous sites that underwent positive selection in the mammalian lineage, particularly in the transmembrane channel-like domain (Fig. 3; Additional file 1: Table S2). In fact, one PSS is located in the transmembrane domains S2, S3, and S4 and two PSSs in the proposed location of the MET channel pore [40], likely indicating that these key functional regions were remodeled as a consequence of positive selection (Fig. 3A; Additional file 1: Table S2). The rest of the PSSs are located in the cytoplasmic and extracellular linker regions (Fig. 3).

We also found that the key IHC gene *otoferlin* (*OTOF*) carries multiple PSSs (Additional file 2: Fig. S2 [41];). OTOF has been described as composed of six or seven C2 domains, one Fer1 domain, and one FerB domain [41, 42]. Calcium binds the C2 domains, giving otoferlin the possibility to act as a calcium sensor and to mediate exocytosis [42]. Hence, the role of these C2 domains in the synaptic transmission at the ribbon synapse of the IHC has been intensely studied [41, 42]. However, only one of the PSSs found in this study is located in a C2 domain (Additional file 2: Fig. S2; UniProt: Q9HC10-1, site 1236), while the rest of them are dispersed in the linker regions. In addition, three of these PSSs have been reported to contain flagged SNPs in humans: rs397515600, rs199904558, and the pair rs145019640/rs80356590. The role of these linker regions in the function of the protein, and its relevance in the evolution of hearing, is yet unknown.

It is also noteworthy mentioning that *LOXHD1*, an understudied protein with the highest number of PSSs (68 sites), has several characteristics that make it relevant for mammalian evolution (Additional file 1: Table S2; Additional file 2: Fig. S3). We found that most of the sites are located in the PLAT (polycystin/lipoxygenase/α-toxin) domains of LOXHD1 (Additional file 2: Fig. S3). PLAT domains are believed to be involved in targeting proteins to the plasma membrane. In fact, this protein localizes in the stereocilia membrane in mature mechanosensory hair cells, in both inner (IHC) and outer hair cells (OHC) [43]. First, in mammals, the maturation of the stereocilia bundle in the Organ of Corti is correlated with the loss of the apical kinocilium. A peak of *LOXHD1* expression is observed around the time of loss of the kinocilium, and it has been suggested that this protein might stabilize the stereocilia in the absence of kinocilium [43]. Second, distortion product otoacoustic emissions were absent in mice mutants (*Samba*) for this gene [43]. This implies that the observed deafness phenotype is in part due to the protein affecting OHC function [43], a cell type unique to mammals. It is likely that the several PSSs found in this study contributed to shape this protein function in mammals. However, to assess this hypothesis, the interaction of this protein with other members of the stereociliary bundle needs to be further studied.

In order to predict the functional impact of amino acid variations occurring in the mammalian lineage, we run PROVEAN [44] on positively selected sites. In this approach, amino acid variants which deviate from the frequently occurring residues are predicted as deleterious to protein function. We identified various positive selected sites in several genes that could have impacted protein function (Additional file 1: Table S6). Background species variants detected over positively selected sites in *CDH23*, *ELMO3*, *LOXHD1*, *OTOF*, *RNF10*, and *SCRIB* would have a deleterious impact on modern human proteins. This could account as a proxy for evolutionary relevant sites that could also have an impact in the clinic, such as two positively selected sites in LOXHD1: 833 R, which corresponds to flagged SNP rs188119157 and 785 A, which is flagged SNP rs886042940 (Additional file 1: Table S6). However, functional studies will be necessary in order to evaluate the clinical impact of these variants.

### Extensive analysis of positive selection in the human lineage

One of the biggest open questions about the evolution of humans is how language evolved and in that sense, how the auditory system was modified in order to serve this human capacity. It has been hypothesized that a co-evolutionary process occurred between human speech and hearing, and as a consequence, the human cochlea developed certain characteristics in parallel to the acquisition of spoken language. Anatomical comparisons of the length of different hominin fossils have allowed us to infer that early hominin taxa show a heightened sensitivity to frequencies between 1.5 and 3.5 kHz and an occupied band of maximum sensitivity that is shifted toward slightly higher frequencies compared to chimpanzees [45] that display heightened sensitivity between 1 and 2 kHz. Thus, early hominin auditory patterns may have allowed short-range communication in open habitats, but probably did not involve the transmission of information beyond that of chimpanzees. Therefore, early hominin’s communication was probably restricted to the frame stage of speech production, a phoneme-based, presyntactic form of communication with only limited word formation. In contrast, *H. sapiens* shows a broad region of heightened sensitivity between 1 and 5 kHz and a wider bandwidth of maximum sensitivity that is extended toward higher frequencies. This wider bandwidth most probably facilitated specialization in the use of complex, short-range vocal communication, including high-frequency consonant production and increased word formation [46]. In addition, using computerized tomography scans and an auditory model other scientists have calculated the occupied bandwidth in Neanderthals and found that this was similar to extant humans, implying that Neanderthals evolved the auditory capacities to support a vocal communication system as efficient as modern human speech [47]. Given that genes related to oral language have been found to be under positive selection in their coding and non-coding sequences [48–52], we aimed to investigate if signatures of positive selection are also present in hearing loss genes, as mutations in their sequences are a prevalent cause for communication disorders. We took our initial dataset of HL genes and explored signatures of positive selection in the human lineage with two approaches. First, we compared human sequences against a background of up to 25 primate sequences. Using *codeml*, we found that 6 genes show signatures of positive selection in the human lineage (Table 2; Additional file 1: Table S3). Among them it is noticeable the presence of *MYO6*, a gene whose function in the inner ear has been very well characterized [53, 54], as one of the key molecular motors involved in stereocilia development [55]. In addition, the gene *ESRRB*, encoding estrogen-related receptor beta is expressed during inner-ear development and has been identified in autosomal-recessive nonsyndromic hearing impairment [56]. Noticeably this gene also displays signatures of positive selection in the mammalian lineage (see below). Other genes have been only recently reported as causative of hearing loss in humans or mice like *MYH9* and *SLC4A10* [57, 58], with the former being previously detected as positively selected [59, 60] and the latter holding archaic introgression as part of the Solute Carrier (SLC) family [61]. On the other hand, some of these genes displaying signatures of positive selection in the human lineage have not been completely characterized in their specific function in the inner ear (*ATP2B1* and *MYO10A*). While other members of the myosin family were regarded as having at least one type of human-specific selection [62–65], an interesting candidate is *MYO10A*, an unconventional myosin that produces a Waardenburg-like syndrome when deleted in mice [66].

**Table 2:** Genes with signatures of positive selection in the human lineage

Positively selected hearing loss genes in the human branch	
*ATP2B1, ESRRB, MYH9, MYO10, MYO6, SLC4A10*	

However, genomic comparisons between species alone do not inform us about ongoing and recent selection within humans, and have little weight if genes have been influenced by only a single, recent selective event [67]. In order to detect such recent selection events, population genetic data are needed. To complement our initial search, we aimed to find signatures of selective sweeps in these genes using the 1000 genome selection browser [68]. We also intersected data from the PopHumanScan database that compiled genomic regions showing signatures of positive selection in human populations [69]. We found that the gene *FMNL3* displayed high values of summary statistics that are based on the site frequency spectrum such as Fu and Li’s D and F, whereas the gene *C12orf42* displayed signatures related to linkage disequilibrium such as XP-EHH (Additional file 1: Table S3). At the same time, we used previously published databases of ancient positive selection [70] to characterize surrounding regions that could have been under selective pressures. We found that only one gene, *SLC4A10*, that displayed signatures of positive selection in the human branch, also shows evidence of ancient positive selection in humans [70]. In this database, we found several genomic regions including the hearing loss genes *KLHL18*, *FNBP1L*, *SLITRK1*, *TRIM71*, and *CXCL3* displaying signatures of ancient positive selection in humans. *CXCL3* was also found to be under recent positive selection [65]. However, none of these genes showed a signal of positive selection in their coding regions when analyzed with PAML *codeml* in our study. Since there are not many studies evaluating the evolutionary characteristics of human hearing, more evidence is needed to validate the relevance of the detected selection signals on hearing loss genes relevant in the physiology of our inner ear. The genes identified here constitute a starting point to unravel the genetic bases of the evolution of hearing in the human lineage.

### Noncoding conserved regions analysis

As an additional possibility, evolution could be acting by modifying regulatory regions impacting on the rate and pattern of expression of hearing genes. To explore potential candidates, we identified non-coding regions with a deviation from the non-neutral substitution rate using databases of acceleration in mammals (ncTSARs [26];) and humans [non-redundant Human Accelerated Regions (HARs) [71];]. Our analysis indicated a total of 14 genes containing at least one ncTSAR element in their transcriptional unit (Fig. 1; Additional file 1: Table S4). Of these 14 genes, only one *Diaphanous homolog 3* (*DIAPH3*) showed both signatures of acceleration in conserved noncoding regions and positive selection in the coding regions. The non-coding accelerated element, TSAR.1094, is located in between *DIAPH3* exons 8 and 9 (chr13: 59,912,022-59,912,393), and there is no previous evidence of experimental regulatory function in the literature. Most of the genes had one TSAR located in its introns (Additional file 1: Table S4) except for *Mirror-Image Polydactyly 1* (*MIPOL1*) with two TSARs (TSAR2 840 and TSAR0923) and *Exocyst Complex Component 4* (*EXOC4*) with three TSARs (TSAR1392, TSAR2949, TSAR3197). Overall, these results indicate that the number of genes displaying signatures of noncoding evolution in the mammalian branch is lower than the number of genes showing signatures of adaptive evolution in their coding regions. We further corroborated this observation by exploring how many accelerated elements intersected HL gene regulatory domains and found a lower proportion for genes with noncoding evolution (Additional file 1: Table S10).

We observed that 23 hearing loss transcriptional units displayed 27 HARs in conserved noncoding regions (Fig. 1; Additional file 1: Table S4). Twenty-one of these genes had one HAR, whereas the gene *ADAMTS9* (*ADAM Metallopeptidase With Thrombospondin Type 1 Motif 9*) presented three HARs and the genes *TSPEAR* and *ROR1* (receptor tyrosine kinase-like orphan receptor 1) displayed two HARs each (Additional file 1: Table S4). In short, these results show that the number of genes showing noncoding accelerated evolution in humans exceeds largely the counterpart displaying coding positive selection. In order to identify evolutionary forces acting into these HL-associated accelerated noncoding regions, we explored the influence of GC-biased gene conversion [71, 72]. None of the reported regions showed signatures of GC-biased gene conversion. Only one HAR (HAR125; linked to the gene *ELMO1*) had conflicting results showing evidence of both positive selection and low confidence GC-biased gene conversion as defined by [72]. Altogether, these results suggest that positive selection is very likely to be responsible for the nucleotidic changes observed in these human-accelerated noncoding regions.

To further corroborate that these regions are subject to positive selection in humans, we intersected them with genomic selection signatures from human populations [68, 69]. Again, several genes were under selection (*ROR1*, *VTI1A*, *AFF3*, *ACVR1*, *ADAMTS9*, *ICK*, *ELMO1*, *EXOC4*; Additional file 1: Table S4). Interestingly, two out of the three genes displaying multiple HARs showed selection signatures. As an example, *ADAMTS9* displayed signatures of selection in XP-EHH values, indicative of linkage disequilibrium (LD), such as long haplotypes and recent selective events [73]. Another interesting case is the gene *ROR1*, which contains two HARs, since it showed high values of XP-EHH indicating long haplotypes in CEU populations and also high values of summary statistics that are based on the site frequency spectrum such as Fu and Li’s D and F.

### Analysis of functional evidence of the regulatory function of ncTSARs and HARs

In order to assess the putative regulatory function of ncTSARs and HARs linked to HL genes, we explored whether there was evidence of regulatory function in published literature and functional genomics consortia. Numerous ncTSARs and HARs present in HL genes have signals of DNAse I hypersensitive sites (Additional file 1: Table S5) compatible with regulatory function [Schema for DNase Clusters - DNaseI Hypersensitivity Clusters in 125 cell types from ENCODE (V3) [74];]. Some of them hold epigenetics marks compatible with regulatory function according to data from the OregAnno database [75] and the GeneHancer Regions database [76]. In contrast, no regions overlapped regulatory elements from the VISTA Enhancer Browser database of transgenic mice [77]. Two non-coding accelerated elements overlapped prosensory-specific otic enhancers (TSAR.1577 and TSAR.4260; Additional file 1: Table S5), which were detected by ATAC-Seq in Sox2-positive cells [78]. A recent work inspected the methylome of the mouse inner ear at several developmental and adult stages [79]. In this work, the authors described unmethylated regions (UMRs) and low methylated regions (LMRs). UMRs are regions with an average methylation lower than 10% and are mostly localized in transcription start sites. On the other hand, LMRs display an average methylation between 10% and 50% and are predominantly located far from TSS in intergenic or intronic regions and are then considered distal regulatory elements. Since these LMRs mostly (84.5–90.2%) overlapped known H3K4me1 sites from the mouse ENCODE project it has been proposed that they probably function as enhancer elements, and thus, we used this information to characterize our noncoding elements. We found that 13 out of 47 (27%) ncTSARs and HARs linked to HL genes overlapped LMRs indicating that they probably behave as enhancers in the sensory epithelium of the mouse developing inner ear (Additional file 1: Table S5). Overall, a significant proportion of inner ear transcriptional enhancers linked to HL genes were shaped by non-coding accelerated evolution in the mammalian and human lineages.

### Enhancer assays of non-coding accelerated regions in transgenic zebrafish

We tested the ability of seven selected ncTSARs to act as transcriptional enhancers during development in a reporter expression assay in transgenic zebrafish (Additional file 1: Table S9). Enhancer assays in transgenic zebrafish are a useful tool to identify regulatory regions even in the absence of sequence conservation in these species [80]. In fact, this strategy has allowed the identification of functional enhancers for human or other mammal’s genes [50, 81–83] using both stable and transient expression assays. To generate transgenic zebrafish, we cloned the conserved region containing each of these selected ncTSARs upstream of the mouse *cFos* minimal promoter fused to the reporter gene *EGFP* (Additional file 3: Table S11; Additional file 1: Table S9). The elements were injected into wild-type AB strain zebrafish embryos.

We chose to study *DIAPH3*-TSAR.1094 because, in our analyses, the gene *DIAPH3* was the only one displaying signatures of positive selection in both coding and noncoding regions in the mammalian lineage. We generated five stable transgenic lines and analyzed them at 24, 48, and 72 hpf. Our analyses showed that *DIAPH3*-TSAR.1094 drove reproducibly *EGFP* expression patterns, mainly to different domains of the developing central nervous system, including the forebrain, hindbrain, and optic tectum. In addition, we observed strong expression at the otic vesicle, heart, and somite muscles at the developmental stages analyzed (Additional file 2: Fig. S4 and Additional file 3: Table S11). This noncoding TSAR did not show any epigenetic mark in cells and tissues (Additional file 1: Table S5). A mutation in the *DIAPH3* gene is responsible for autosomal dominant nonsyndromic auditory neuropathy 1 (AUNA1 [84–86];). *DIAPH3* belongs to the formin-related family, known to promote the nucleation and elongation of actin filaments and to stabilize microtubules [87]. Strikingly, a point mutation in the 5′ untranslated region of the human *DIAPH3* leads to overexpression of the DIAPH3 protein [88]. *Diap3* (the murine ortholog of *DIAPH3*) is strongly expressed in IHCs and OHCs in the adult mouse [89]. Transgenic mice overexpressing *diap3* have been a useful tool to dissect the AUNA1 mechanism [90], demonstrating that overexpression of diap3 in transgenic mice recapitulates the human AUNA1 phenotype, i.e., a delayed-onset and progressive hearing loss leaving OHCs unaffected [90]. In sum, we found that *DIAPH3* underwent extensive evolutionary remodeling and the noncoding region *DIAPH3*-TSAR.1094 behaved as a gene expression regulatory region.

We also explored in transient and stable enhancer assays two out of the three accelerated noncoding regions associated with the gene *EXOC4*: EXOC4-TSAR.1392 and EXOC4-TSAR.2949. We chose the *EXOC4* gene and its associated elements as representatives of a gene with unknown function in the inner ear but with three candidate accelerated elements (representing one of the transcriptional units with the highest accumulation of noncoding accelerated regions). We found that EXOC4-TSAR.1392 showed expression in the otic vesicles, heart, blood cells, and forebrain in transient expression analysis (Additional file 3: Table S11 and Additional file 2: Supp. Fig. S5 [91];). On the other hand, EXOC4-TSAR.2949 did not show expression in stable transgenic zebrafish assays at the developmental stages analyzed (Additional file 3: Table S11; Additional file 2: Supp. Fig. S5). The region EXOC4-TSAR.3197 not explored through transgenic assays displays signatures of functioning as a transcriptional enhancer at mouse postnatal day 22 (P22) in methylation assays [79]. We have not found previous reports about the function of this gene in the inner ear beyond the report generated by the IMPC that heterozygous mouse carrying mutations in this gene display aberrant ABRs at 24 kHz (MGI:1096376 [22];). In addition, expression analysis in the mouse cochlea at E16.5 [92] and in adults [93] showed that this gene is expressed in the developing and adult inner ear (Additional file 2: Supp. Fig. S5). Thus, the expression in the developing inner ear displayed by EXOC4-TSAR.1392 and the low methylation marks found in EXOC4-TSAR.3197 in the mouse inner ear could indicate that in fact these regulatory elements could be controlling the expression of this gene in this organ. Further studies will be necessary to assess the function of this gene in the inner ear. It is interesting to note that whereas no signals of positive selection and/or acceleration were found in the coding regions of this gene, three TSARs and one HAR (ANC1203) are linked to *EXOC4* (Additional file 1: Table S5) suggesting that the regulatory machinery of this gene has been extensively modified not only in the mammalian but also in the human lineages.

We cloned and generated six stable transgenic zebrafish lines carrying *SMOC1*-TSAR.3685 that show expression in the nervous system including the hindbrain, midbrain, forebrain, spinal cord, eyes, and also show expression in the developing ear and the heart muscle (Additional file 2: Fig. S6 and Additional file 3: Table S11 [94];). *SPARC Related Modular Calcium Binding 1* (*SMOC1*) encodes a multi-domain secreted protein that may have a critical role in ocular and limb development. According to the phenotyping performed by the IMPC it is lethal beyond weaning and mouse carrying mutations display several skeletal defects (MGI:1929878 [22];). This gene has previously been associated with anophthalmia and microphthalmia [95] but not to inner ear function, except by the report generated by IMPC that heterozygous mice carrying *SMOC1* mutations display abnormal ABR recordings. The GeneHancer regulatory element that encompasses *SMOC1*-TSAR.3685 shows evidence of interacting with the SMOC1 promoter, and in addition, this element displays H3K27ac epigenetic marks that indicate regulatory function (Additional file 2: Fig. S6).

We also analyzed four stable transgenic lines carrying *MIPOL1*-TSAR.2840, which show expression in the nervous system including the hindbrain, midbrain, forebrain, eyes, otic capsule, and the heart. While there are some enhancers in the *MIPOL1* locus already associated with the *MIPOL1* promoter (Additional file 2: Fig. S7 and Additional file 3: Table S11), the element *MIPOL1*-TSAR.2840, in particular, has not been tested previously. We observed that the four lines analyzed showed strong wide expression at 24 hpf whereas the pattern of expression was narrowing to more specific structures at 48 and 72 hpf. In three of the four lines, we observed expression in the otic capsule (Additional file 2: Fig. S7). It is interesting to note that *MIPOL1*-TSAR.2840 displays low methylation signal in the developing and adult ear in mice [79]. *MIPOL1* function in the inner ear is still unknown, although as reported by IMPC, a mutant mouse carrying mutations in this gene displays aberrant ABRs at 24 kHz (MGI:1920740 [22];). However, expression analyses show that this gene is in fact expressed in the inner ear of mice and also in different cell types of the developing lateral line in zebrafish (Additional file 2: Fig. S7, C [93, 96];).

We also studied an intronic element (*GATA2*-TSAR.3936) located in the *Gata2 locus*, as *Gata2* has essential roles in the development of many organs and during mouse inner ear morphogenesis, where it is expressed in the otic-vesicle and the surrounding periotic mesenchyme [97]. This transcription factor is critically required from E14.5-E15.5 onward for vestibular morphogenesis [98] and also function redundantly with GATA3 to maintain spiral ganglion cells and hearing [99]. In particular, the element *GATA2*-TSAR.3936 shows evidence of being a *GATA2* enhancer (TSAR.3936:OREG0002949). We generated four stable transgenic lines and our results indicated that *GATA2*-TSAR.3936 drives *EGFP* to the developing nervous system particularly to the hindbrain, midbrain, and spinal cord in transgenic zebrafish at the three stages analyzed (Additional file 2: Fig. S8 and Additional file 3: Supp. Table S11). This accelerated element drives also strong expression of the reporter gene to the otic capsule at the three stages analyzed (3 out of 4 lines analyzed). In situ hybridization studies of gata2a (Additional file 2: Fig. S8) show that this gene is expressed in the cranial region, hindbrain, tegmentum, otic vesicle, eye, and pharyngeal arches in the developing zebrafish [100].

**Fig. 4:**
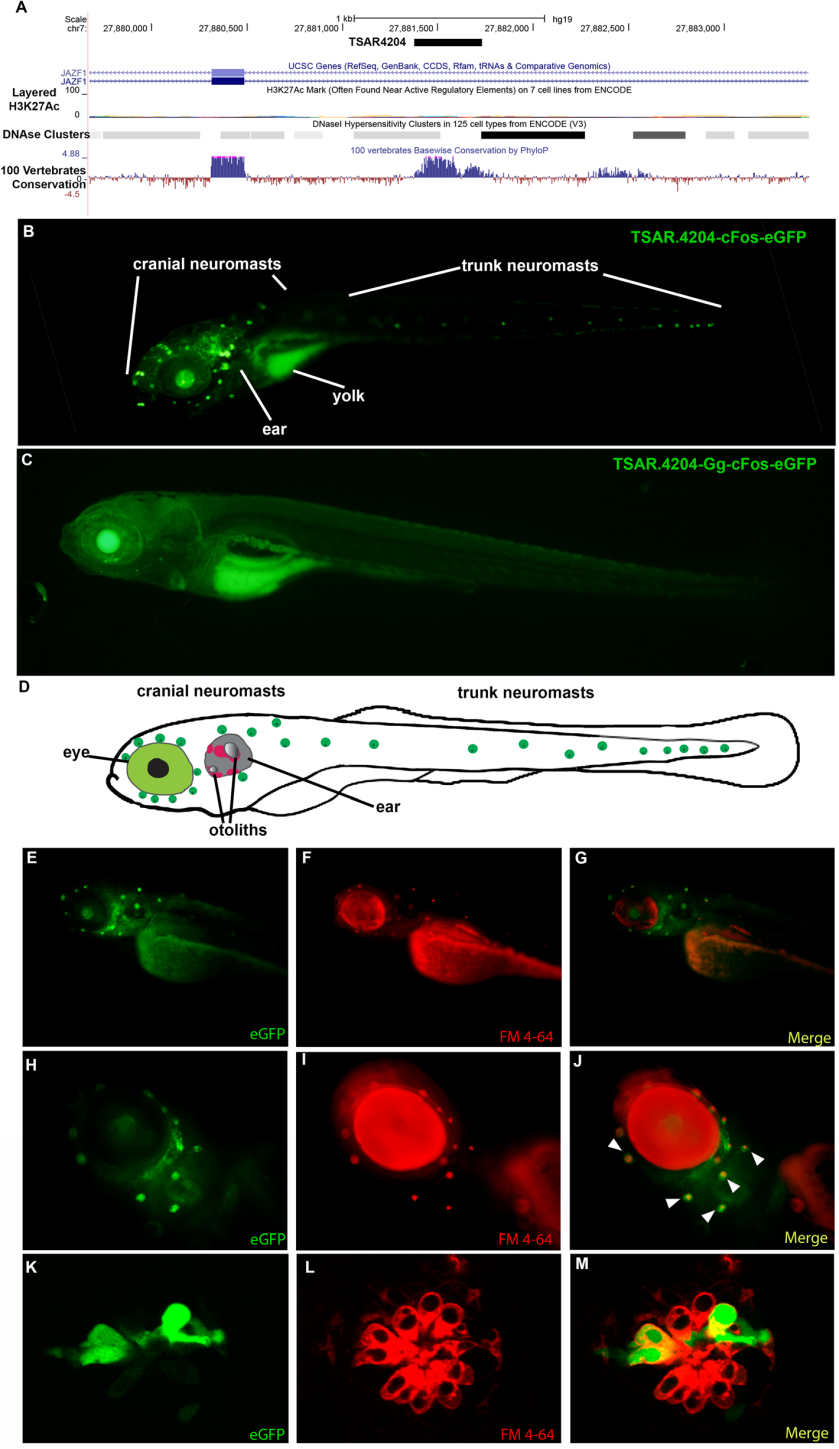
Representative transgenic zebrafish carrying the TSAR4204-JAZF1. **A** Location of the TSAR.4204 in the JAZF1 locus in chromosome 7 of the human genome (modified from UCSC Genome Browser). **B** Low-magnification microphotograph showing the expression pattern of the transgene TSAR.4204-cFos-eGFP (human sequence as a mammalian representative) in a transgenic zebrafish at 6 dpf. **C** Low-magnification microphotograph showing the expression pattern of the transgene TSAR.4204-Gg-cFos-eGFP carrying the chicken ortholog of the TSAR.4204 sequence in a transgenic zebrafish at 6 dpf. **D** Scheme of a 6 dpf zebrafish showing the approximate location of the inner ear and neuromasts of the lateral line, where the transgene carrying the mammalian sequence of TSAR.4204 directs the expression of eGFP. Figures (**E**–**M**) show magnifications of the head region of a transgenic zebrafish carrying the human sequence of TSAR.4204-cFos-eGFP exhibiting in major detail the expression of eGFP (**E**, **H**, **K**), the fluorescent marker FM4-64 that specifically labels hair cells (**F**, **I**, **L**), and a merged image showing the colocalization of eGFP and the hair cell marker (**G**, **J**, **M**). Figures (**K**–**M**) show in detail confocal images of neuromast expressing eGFP in the lateral line of transgenic zebrafish carrying the human sequence of TSAR.4204-cFos-eGFP. We show the best representative image for each line

Finally, we studied a noncoding element located in the *JAZF1* locus, a gene without previous evidence of function in the inner ear. We analyzed seven stable transgenic lines carrying the human version of *JAZF1*-TSAR4204 (*JAZF1*-TSAR4204-Hs) that displayed a strong expression pattern of the reporter gene in the otic capsule, eye, developing nervous system, heart, and somitic muscles at 24, 48, and 72 hpf (Additional file 2: Fig. S9 and Additional file 3: Supp. Table S11). Then, starting at 48 hpf and specially at 72 hpf, we observed strong expression of *EGFP* in the head and trunk neuromasts of the lateral line (Additional file 2: Fig S9 and Additional file 3: Table S11). We continued to analyze the expression in the lateral line at 6 days post fertilization (dpf) since this system is well developed at this stage and found that *EGFP* was strongly expressed in the neuromast. We also found that the reporter gene colocalized with the hair cells marker FM4-64 suggesting that *JAZF1*-TSAR4204-Hs specifically directs the expression of the reporter gene to hair cells in this sensory system (Fig. 4 and Additional file 2: Fig. S10). The neuromasts are composed of receptive hair cells and are part of the lateral line system of fish, which in turn is a mechanoreceptive organ that enables mechanical modifications of the water to be sensed. Although the lateral line organ is not present in mammals, the morphology and function of sensory hair cells are evolutionarily conserved from fish to mammals [101, 102]. It has been shown that lateral line and ear hair cells develop and differentiate by similar developmental mechanisms and also that mutations in genes causing deafness in humans also disrupt hair cell function in the zebrafish lateral line and vestibular system [103].

To compare the *JAZF1*-TSAR4204 mammalian pattern of expression to an outgroup, we cloned the orthologous *JAZF1*-TSAR4204 region from chicken (*JAZF1*-TSAR4204-Gg) and generated three stable transgenic zebrafish lines to test its enhancer function. We found that *JAZF1*-TSAR4204-Gg does not function as an enhancer in the lateral line neuromasts whereas expression is conserved in other organs such as the nervous system (Fig. 4C; Additional file 2: Fig S9) suggesting that *JAZF1*-TSAR4204 could have gained enhancer function in these hair cells due to the evolutionary process that this genomic region underwent in the mammalian lineage. Transcriptional data indicates that *JAZF1* is expressed in the cochlea of adult and developing mice (Additional file 2: Fig. S9). In addition, the two orthologs of JAZF1 in zebrafish (Jazf1a and Jazf1b do not express differentially in hair cells of the developing neuromasts of the lateral line (Additional file 2: Fig. S9)). However, this is only a first approximation to the study of *JAZF1*-TSAR4204 function in the inner ear, which will benefit from the incorporation of other approaches, including chromatin conformation capture, gene editing, and also additional studies in a mammalian model. Together, these studies could confirm if TSAR4204 indeed regulates *JAZF1*. In the meantime, our findings suggest that *JAZF1*-TSAR4204 could behave as a specific enhancer region directing the expression of the regulated gene to hair cells and that this function was gained in the mammalian lineage.

In summary, zebrafish transgenic assays allowed us to find that most of the elements with signatures of accelerated evolution are transcriptional enhancers (6 out of 7). Moreover, the majority of them directed expression to the developing inner ear. Interestingly, we found that the mammalian ortholog of JAZF1-TSAR4204 possibly gained function as a hair-cell-specific enhancer during evolution, since the chicken ortholog does not direct expression to hair cells of the lateral line neuromasts. Experiments on mammalian animal models will help to elucidate whether this regulatory role translates into a precise activity in hair cells in the organ of Corti.

### Gene functions and network interactions

We were curious to explore if there was any significant gene ontology enrichment for the different sets of genes analyzed in this study. First, we evaluated the characteristics of our compiled hearing-loss dataset. The HL genes are overrepresented in protein binding, ion binding, catalytic activity, organic cyclic compound binding, heterocyclic compound binding, and others (Additional file 1: Table S7). The top cellular component terms were in terms related to organelle and cytoplasm structures, and the biological processes overrepresented were sensory perception of sound and sensory perception of mechanical stimulus (Additional file 1: Table S7). In the same fashion, we explored if the genes displaying mammalian positive selection had any particular term enrichment. Coding-selected genes were also overrepresented in protein binding and ion binding, but, in contrast, also in cytoskeletal protein binding and actin binding (Additional file 1: Table S7). Most mammalian positively selected genes are involved with cytoskeletal and actin binding molecular functions whereas these categories are not among the top functions in our HL database. In line with these findings, the top cellular component terms among positively selected genes in mammals were organelle, stereocilia bundle, and cluster of actin-based cell projections (Additional file 1: Table S7). These results suggest that approximately 210 million years ago, genes that putatively code for acting-binding or actin-remodeling underwent selection in their coding portions, a finding consistent with the hypothesis that the actin-based structural shape of the mammalian inner ear is necessary for normal hearing [104, 105].

The group of genes displaying signatures of acceleration in noncoding regions (ncTSARs) shows striking differences to those showing mammalian positive selection in their coding regions (Additional file 1: Table S8; Additional file 1: Fig. S11). In effect, cis-regulatory region sequence-specific DNA binding and transcription regulator activity appear among the top molecular function GO terms when we analyzed ncTSARs carrying genes whereas coding-selected genes show overrepresentation in calmodulin binding, cytoskeleton binding, actin binding, etc. (Additional file 2: Fig. S11). Furthermore, among biological functions related to ncTSARs, sensory perception of sound was not present but, instead, regulation of cellular processes and development. Moreover, the cellular component terms were never related to stereocilia actin-bundle. Our results indicate that coding and noncoding selection-shaped genes in the mammalian lineage play different functions are involved in different processes and are located in different compartments of the cell. In summary, our analyses suggest that genes displaying signatures of positive selection in noncoding regions are involved in regulation of expression whereas genes that underwent positive selection in coding sequences are related to specific hair cell functions such as mechanotransduction as evidenced by the overrepresentation of GO terms related to cytoskeleton function.

Regarding genes displaying signatures of acceleration/selection in the human lineage, we could not analyze genes displaying positive selection in coding regions due to the small number of genes in this group. On the other hand, genes associated with HARs are enriched in protein kinase activity and transmembrane receptor functions (Additional file 1: Table S8). We compared if genes associated with accelerated noncoding regions in the human and in the mammalian lineage were linked to different functions, biological processes, or cellular compartments. We found that these two groups of genes have in common some molecular functions but also each group has unique tasks. In fact, fewer genes linked to HARs are associated with transcription factor activity or regulatory activity in clear contrast to genes associated with ncTSARs. Alongside, ncTSARs are associated with RAS GTPase binding whereas HAR genes are associated with protein kinase activity (Additional file 1: Table S8 and Additional file 2: Fig. S12). On the other hand, both groups of genes are connected to ion and heterocyclic compound binding. Regarding the cellular component, HAR-linked genes play their function in the cytoplasm, associated to membrane-bounded organelle, cell projection, cell junction, and whereas TSAR-linked genes do not show association to these cellular compartments. Our results indicate that some functions have been shaped by accelerated evolution in noncoding regions in both the mammalian and the human lineages whereas some others were exclusively impacted in each particular lineage.

**Fig. 5:**
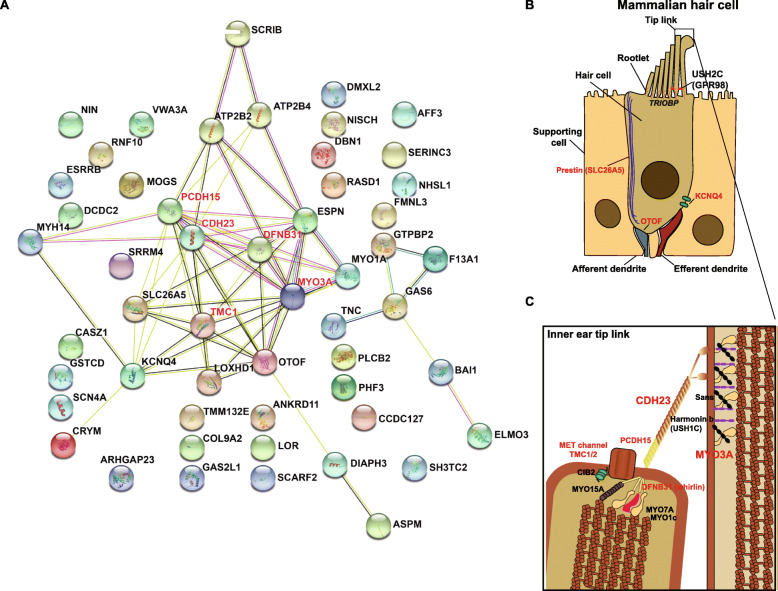
Key genes involved in the mechanotransduction machinery display signatures of positive selection. **A** A network interaction map of all the 53 positively selected proteins from the STRING database (https://string-db.org). Edges represent protein associations, with either known or predicted interactions. Known interactions are represented by light-blue lines (curated databases) or purple lines (experimentally determined). Predicted interactions are represented by green (neighbors), red (fusions), and blue (co-occurrence) edges. Other interactions are represented by gold (text-mining), black (co-expression), and gray (protein-homology) lines. Interaction is observed between classical, well-known hearing genes (*PCDH15*, *CDH23*, *TMC1*) related to the mechanotransduction machinery and novel genes (*LOXHD1*), for which its role in this complex has yet to be explored but still show strong signals of positive selection. Genes involved in the tip link function are highlighted in red (**B**). Cellular organization of the mammalian hair cell. Genes that play key roles in hair cell function and displayed signatures of positive selection in mammals are highlighted in red. **C** Tip link schematic showing the location of key genes involved in function in this particular structure. Genes involved in the mechanotransduction machinery that display signatures of positive selection are highlighted in red

The 53 mammalian positively selected genes were also inspected for network association using the STRING database. This database collects information about known and predicted protein-protein interactions, which include direct (physical) and indirect (functional) associations. In the interaction map, it is possible to observe that several genes form a densely connected hub with key hair cell genes such *SLC26A5* (encoding Prestin, the motor protein of outer hair cells), *MYO3A*, *OTOF*, *GPR98*, *LOXHD1*, and *DFNB31* (Fig. 5A). In particular proteins that are localized in the tip link: *PCDH15*, *CDH23*, *TMC1* (Fig. 5B, C) showed, as expected, strong interaction evidence (Fig. 5A). It has been demonstrated that *PCDH15* and *CDH23* interact to form tip links where they localize to the lower and upper parts, respectively (Fig. 5B, C) [106–108]. It is noticeable that *DFNB31* recently identified as the usherin *type 2 gene* (*USH2*) encoding whirlin (*WHRN*) displays numerous interactions with *PCDH15*, *CDH23*, *TMC1*, and *MYO3A* [109–111]. Outside of the hub, interaction evidence was found for *DIAPH3* and *ASPM*; *PLCB2*, *MET*, and *BAHD1* (Fig. 5A). A smaller cluster of genes was composed of *GTPBP2*, *GAS6*, *F13A1*, *THB51*, *TNC*, *COL11A1*, *LEPRE1*, *BAI1*, and *ELMO3*. On the other hand, our analysis shows that there are several genes outside of the hub that do not display any evidence of interaction (Fig. 5A) suggesting that many of these genes are still unexplored and their functions and roles in the inner ear are poorly understood.

This analysis also revealed only a few genes displaying signatures of noncoding acceleration with connections to other genes (Additional file 2: Fig. S13, C). As highlights we can mention *JAZF1* and *ADAMTS9*, which in turn interacts with *CDH23*. Nevertheless, functional relationships among these three genes in the inner ear have not been reported yet. Another link between *CDK14* (*Cyclin Dependent Kinase 14*) and *ICK* (*Intestinal Cell Kinase*) was evidenced by this analysis, although, not previous report of *CDK14* function in the inner ear was found, it has been recently reported that *ICK* plays an essential role for planar cell polarity formation in inner ear hair cells [112]. In addition, there was evidence of interaction among *GATA2*, *KITLG*, and *ACVR1* and also between *EPHA5* and *TSPEAR*. The last two genes constitute interesting cases for follow-up studies since both have been uncharacterized regarding functions in the inner ear. Whereas *EPHA5* has been shown to be expressed in the developing inner ear [113], its function has been only characterized during visual system development where it regulates patterning of the topographic connections [114, 115]. *TSPEAR* encodes the thrombospondin-type laminin G domain and EAR repeats protein, and its function is poorly understood, although it has been recently shown that plays a critical role in human tooth and hair follicle morphogenesis through regulation of the Notch signaling pathway [116].

On the other hand, our STRING interaction analysis for genes displaying signatures of acceleration in coding regions show a dense hub of interacting genes including *CDH23*, *MYO15A*, *PTPRR*, *GRXCR1*, *GPR98*, *USH1C*, *MYO1A*, *TECTA*, *COL11A2*, *DUSP7*, and *OTOG* (Additional file 2: Fig. S13, B). As previously mentioned many of these genes have largely been involved in key functions in the mammalian inner ear; however, *PTPRR* and *DUSP7* have not been functionally characterized in the inner ear and our analysis indicates that they are interesting candidates for follow-up studies.

We compared the intersection of the different sets of genes with evidence of selection (at either their coding or non-coding regions) in the mammalian and the human lineages. The set with the highest number of members was the one containing genes displaying coding positive selection in mammals (Additional file 2: Fig. S13, A). This contrasted with the smaller number of genes associated with ncTSARs. This pattern was replicated when we consider as putative regulatory regions those elements located not only inside the gene body, but also inside a gene regulatory domain (see the “Methods” section and Additional file 1: Table S10). However, this pattern is not observed when we consider all ARs located in chromatin hierarchical 3D structures or topologically associated domains (TADs) as associated with a HL gene. TADs are defined using chromatin conformation data from different cells and tissues and are thought to bring distal regulatory elements in close proximity to the target promoter, but chromatin-capture data is currently unavailable for the inner ear. We therefore approximated this kind of analysis by using data from the human fetal brain [117] and considered that all elements in a TAD could potentially interact with the HL gene promoters. Using this approach, we observed that the number of noncoding changes in HL genes surpassed coding changes, but we have to consider that this latter approach is probably an overestimation, given that not all regulatory elements located in a given TAD could indeed interact with all gene’s promoters included in that TAD (Additional file 1: Table S10). Opposite to this pattern, in humans, there was a higher proportion of genes displaying signatures of selection at non-coding elements (HARs) than genes displaying positive selection in coding regions (Additional file 2: Fig. S13 A). Only one gene displayed signals of positive selection in coding regions in both human and mammalian lineages: *ESRRB*, whereas the genes *EXOC4*, *DIAPH3*, *TOX*, and *JAZF1* showed acceleration in noncoding conserved regions in both lineages. These data suggest that some genes are recurrently the target of selection in different lineages.

## Discussion

In this work, we explored to which extent coding and noncoding changes have been part of the functional evolution of hearing genes that might underlie the emergence of the distinctive characteristics of the mammalian inner ear. First, we evaluated whether coding regions of hearing loss genes, which play a critical role in the development and physiology of the mammalian inner ear, have been targeted by evolutionary forces in both the mammalian and the human lineage and we identified 53 and 6 genes that underwent positive selection, respectively. Second, we also estimated to which extent conserved noncoding regions linked to HL genes were also shaped by lineage-specific evolution. We detected 14 transcriptional units that underwent accelerated evolution in conserved non-coding elements in the mammalian lineage and 23 in the human lineage. Our quest will help to decipher the overall role of coding and noncoding regions that together constitute the functional evolutionary scaffold of genes involved in development and disease of the inner ear.

### Role of coding regions of hearing loss genes in mammalian inner ear evolution

There is a particular relevance of studying genes involved in human disease to unravel inner ear evolution. It has been previously shown that evolutionary rates are lower in genes related to muscular, sensory (including hearing), skeletal, and cardiovascular diseases [118]. Purifying selection is then the most frequent pathway to preserve biological function and acts against deleterious mutations. However, a mutation can also be beneficial and then be fixed by positive selection in a particular lineage. Hence, the discovery of genes related to hearing loss that evolved under positive selection in a given lineage opens the door to untangle the evolution of functional and morphological novelty in the inner ear. A few evolutionary studies have been carried out on genes that are important for mammalian hearing mechanisms, particularly those genes identified as related to non-syndromic hearing loss. On a previous study, Kirwan and collaborators surveyed a few selected hearing loss related genes (*Myo15a*, *Ush1g*, *Strc*, *Tecta*, *Tectb*, *Otog*, *Col11a2*, *Gjb2*, *Cldn14*, *Kcnq4*, *Pou3f4*) [7]. In the mentioned study, only three of these genes were found to display signatures of positive selection (*Myo15a*, *Otog* and *Tecta*). Due to differences in experimental design, some of these genes (*MYO15A*, *STRC*, *OTOG*, *COL11A2*, *GJB2*, *TECTB*, and *USH1G*) did not have enough orthologues to be analyzed for signatures of Darwinian selection in our work. On the other hand, we did analyze *TECTA*, *CLDN14*, *KCNQ4*, and *POU3F4*, but we did not find signatures of positive selection in these genes through PAML analysis. It is important to keep in mind that our study and the one carried by Kirwan et al. used different tests of positive selection (M7 vs M8 and M8 vs M8a in Kirwan et al. and branch-site Model A in our study). While models implemented by Kirwan et al. are site models that focus on detecting heterogeneity between sites among a set of mammalian species [119], the models implemented in this work are branch-site-specific tests and were used to detect signatures of molecular adaptation at particular sites of the protein focusing on the mammalian basal lineage [24]. However, it is interesting to note that we did find signatures of accelerated evolution in coding regions of *COL11A2*, *TECTA*, *MYO15A*, and *OTOG* by means of analyzing cTSAR elements. Thus, it is likely that the use of different alignment methods and tests for positive selection could probably explain the differences between the results found by others and our study. Therefore, the use of two or more approaches combined (coding acceleration via *phyloP*, different alignment methods or positive selection via different PAML models) broadens the detection of lineage-specific evolutionary processes. Among the 53 mammalian positively selected genes identified we found *SLC26A5*, the gene encoding prestin, which has already been reported as an important inner ear gene displaying strong signatures of adaptive evolution in the mammalian lineage [4, 5, 120]. Furthermore, we found correlating signals of positive selection in five hearing loss genes that have been recently identified in a previous study of positive selection of the inner ear transcriptome [6].

In the inner ear, mechanotransduction is thought to occur at the level of stereocilia and/or at the kinocilium at the top of inner hair cells. In this process, it has been shown that tip links, extracellular filaments that connect stereocilia to each other or to the kinocilium, are of fundamental importance [121, 122]. For the development of the stereocilia bundle, *WHRN* (DFNB31) expression is needed. Whirlin is restricted to the tips of stereocilia in mature hair cells [109], and it is involved in the stability of its projections. This protein interacts with the actin cytoskeleton, and we found that the gene encoding for whirlin showed signatures of positive selection in mammals. We have identified two important constituents of the tip link that belong to the cadherin family, *CDH23* and *PCDH15* displaying strong signatures of adaptive selection and multiple positively selected sites. The location of these sites is not randomly distributed: *PCDH15* has positively selected sites in the domains related to the interaction with *CDH23* and with members of the MET channel. In addition, we found that the gene *TMC1*, which has been involved in mechanotransduction in mammalian hair cells and interacts directly with *PCDH15* [36] also displays signatures of positive selection in the mammalian lineage. Recently, *TMC1* was found to be a part of the pore of sensory transduction channels in mechanosensory auditory hair cells [39]. Interestingly, *PCDH15*, *TMC1*, and *CDH23* are among the genes carrying the largest number of positively selected sites. At the same time, studies suggested that the tip link should be attached to a myosin motor to move actin filaments [110]. Our functional protein association network study of positively selected genes identified several members of the myosin family such as *MYO3A* and *MYO1A* displaying evidence of interaction with *TMC1*, *PCDH15*, and *CDH23*. In fact, it has been reported that *PCDH15* interacts with *MYO3A* at the tip link [123]*.* Our results indicated that these molecular motors also displayed strong signatures of positive selection affecting multiple sites, consistent with how sensory hair cells profit from actin in order to be able to exert mechanotransduction [105]. In addition, it has been suggested that in order to repair and strengthen the F-actin core of stereocilia, mammals use an evolutionary conserved assembly and disassembly mechanism [104].

Thus, our study revealed a core of genes strongly interrelated that play a key role in mechanotransduction in hair cells that underwent adaptive evolution in the mammalian lineage suggesting that this crucial hair cell function was evolutionarily remodeled to serve a mammalian-specific function.

### Hearing loss genes underwent non-coding evolution in the mammalian and human lineages

The study of positive selection on coding changes can yield an incomplete depiction of the process of adaptive evolution, which can be improved by studies of accelerated non-coding evolution. It has been suggested that evolutionary changes affecting regulatory regions are more likely to act on morphological evolution since coding changes in developmental genes that usually participate in multiple independent developmental processes can have deleterious pleiotropic effects [12, 124, 125]. We selected several noncoding accelerated regions to perform functional assays in order to assess if they play a gene expression regulatory role. Most of the tested elements behave as transcriptional enhancers during development, directing the expression of the reporter gene to several organs such as the inner ear, lateral line, brain, spinal cord, and heart. The fact that these experimentally characterized active enhancers drove expression to diverse tissues, suggests that regulatory regions display varied levels of pleiotropy as it has been previously suggested [126, 127]. It was already noticed that there are no genes with exclusive expression to inner ear hair cells and that enhancers directing expression to hair cells were not exclusive of this cellular type [11]. Thus, uncovering regulatory regions controlling exclusive and specific expression in hair cells is crucial for developing therapeutic strategies without deleterious effects in other organs or tissues. Therefore, the identification of the regulatory region TSAR.4204 located in one of the introns of the gene *JAZF1* that shows a very specific expression pattern in the inner ear and lateral line of the developing zebrafish, constitutes a relevant finding. A further analysis indicated that only the mammalian ortholog of this element directs the expression of the reporter gene to neuromast’s hair cells of this sensorial system. In contrast, a non mammalian ortholog (chicken) does not direct expression to these tissues, suggesting that this regulatory region gained enhancer function in the mammalian lineage. Since the gene *JAZF1* has never been previously involved in the physiology of hearing, our finding suggests that this gene is an excellent candidate for further studies aiming to unravel its participation in inner ear physiology. The modification of regulatory sequences could potentially alter transcription factor binding sites, contributing to phenotypic diversity within and between species.

### Positive selection in hearing loss genes in the human lineage

A novel perspective that we propose here is that the human cochlea could have evolved in synchrony with speech production. One way to approach this hypothesis is to identify genes related to both hearing loss and developmental dyslexia/language impairment and to study the evolution of these genes on the human lineage. Our scan of positive selection in the human lineage against a background of other primates revealed only a few candidate genes that might have had a role in the evolution of our auditory capacities. *ESRRB* is a gene related to severe bilateral sensorineural hearing loss and, in this study, was found to be under positive selection in both humans and mammals. In addition, *MYH9* was previously detected as positively selected [59, 60] and *SLC4A10* hold archaic introgression as part of the Solute Carrier (SLC) family [61]. We noticed that only one (*SLC4A10*) of the genes identified as positively selected was supported by further selection signatures based on intraspecific polymorphisms, such as selective sweeps. On the other hand, the opposite pattern was found for non-coding human accelerated sequences: the number of HL genes associated with HARs outweighed the number of positively selected genes in the human lineage. Interestingly, the loci comprising HAR regions were more frequently associated with signatures of selective sweeps in human populations. As expected, we found that some HAR-linked genes are related to the regulation of gene expression, possibly acting as developmental enhancers, as it has been reported previously for these accelerated elements [71, 82]. Additionally, several loci that underwent positive selection or acceleration in the mammalian lineage showed selective sweeps in humans. As an example, one of the genes displaying the highest number of mammalian PSSs (*PCDH15*) has been previously identified in several genome wide scans of positive selection in humans [73, 128, 129]. Altogether, these data indicate that key HL genes have been shaped by ancient positive selection in the mammalian lineage but also by more recent processes of adaptive evolution in human populations. Further studies in this field will elucidate if they have truly been important in the adaptation of humans to different environmental set-ups and also to disease resistance across our history.

### Which is the locus for evolution in the inner ear? Perspectives from hearing loss genes

To our knowledge, no “hearing genes” have been detected to arise in a de novo pattern. Therefore, evolution had to work with pre-existing material and modify it with either regulatory or protein modifications. In our analysis about HL genes in mammals, the pattern seems to be biased toward protein-coding adaptations, as they largely exceed non-coding modifications in the mammalian lineage. Additionally, the process of recurrent/ongoing selection and modification of certain proteins is observed, as it has been noticed in other lineages [130]. We could speculate that perhaps hearing loss genes are more specific than the rest of genes expressed in the inner ear and hence, less pleiotropic. In this case, this would allow evolution to play on coding regions without potentially affecting other tissues and now it is reflected as recurrent evolution on several lineages. As an example, the gene encoding for *otoferlin* (*OTOF*) was found to be under signatures of positive selection in the basal branch of mammals (this study), cetaceans [131], and echolocators [132] and showed Fst signals in humans [133]. Interestingly, *OTOF* is a cluster defining gene for IHCs [134]. This finding is curious as selective mechanisms seem to have not only acted on the typical mammalian cochlear amplifier (the OHC), but also in the modification of surrounding cell types as previously suggested [6]. On the other hand, in the human lineage, non-coding evolution is more abundant than coding changes suggesting the existence of different ways to modify either protein function or gene expression at different times throughout history. This pattern is not surprising in evolutionary terms, as there are examples in other organisms where adaptive changes in non-coding DNA are more frequent than their coding counterparts [125] and that the subtle effects of each type of modification combined can lead to complex adaptations [135]. In humans in particular, gene expression divergence was already regarded as a driver for adaptation at a genome-wide scale [136, 137].

## Conclusions

Our study revealed that key genes that participate in mechanotransduction in hair cells underwent adaptive evolution in the mammalian lineage suggesting that this crucial hair cell function underwent functional remodeling in this group of animals probably to serve a specific function. In addition, we identified several noncoding regions linked to HL genes that underwent accelerated evolution in the lineage leading to mammals and function as active developmental enhancers that could underlie the evolution of regulatory changes in mammals. We also found that in the human lineage evolutionary changes in noncoding regions linked to HL genes outnumbered changes in coding sequences and we suggest that these evolutionary driven modifications could have played a role into the emergence of functional changes related to adaptations of the hearing system to the evolution of speech capacity in our lineage.

In summary, our results may suggest that the pathway evolution takes to make morphological and physiological changes involves both coding and noncoding functional sequences, and it might be dependent on the trait under study. Combining approaches from neuroanatomy, comparative biology, paleontology, and evolutionary genetics will lead to new insights into the locus of evolution across divergent tissues and phylogenetic groups.

## Methods

### Non-redundant hearing loss database building

We built our local database of non-syndromic hearing loss genes (HLG) using information from various sources of reported genes that had been linked to deafness and hearing loss. The NSHL database is composed of a total of 115 genes obtained by combining the information available in the “Hereditary Hearing Loss Homepage” [http://hereditaryhearingloss.org [17]], the “Genetics Home Reference – Nonsyndromic deafness related genes” [https://medlineplus.gov/genetics/condition/nonsyndromic-hearing-loss [18]], the Connexin-deafness homepage – Nonsyndromic deafness related connexins' (http://perelman.crg.es/deafness/ [19]), and the chapter “Deafness and Hereditary Hearing Loss Overview” from the online book “Gene Review” [(http://www.ncbi.nlm.nih.gov/books/NBK1434) [20];]. In addition, we used information from the hearing loss screen performed as part of the International Mouse Phenotyping Consortium [(IMPC); (https://www.mousephenotype.org/) [21–23];]. In this study, the authors assessed 3006 mouse mutants for hearing using an Auditory Brainstem Response (ABR) test and identified 330 candidate hearing loss genes. After automated statistical analysis of the data set followed by manual curation, the authors uncovered a set of 67 mutants with robust hearing impairment, in either low or high frequency or across two or more frequencies. Of the 67 genes, 15 were known hearing loss loci, while the vast majority, 52, were novel candidate hearing loss genes that had not previously been associated with hearing loss. Although after manual curation of the ABR data the authors decided that only 67 were considered hearing loss genes, we decided to include in our analysis the complete set of 330 genes that displayed some ABR abnormalities. It has been reported that this kind of high-throughput functional approach in mice could sometimes yield inadequate results for true human hearing loss genes due to the differences in hearing in these two species [138]. We joined all genes present in all the aforementioned databases in order to create a non-redundant hearing loss set of genes (*n* = 431) that we named hearing loss gene database (HLGD; Additional file 1: Table S1).

### Sequences retrieval

All available ortholog coding sequences were downloaded through Ensembl REST API with sequence information from July 2019 (v97). In order to perform the PAML evolutionary analysis on a gene, this must be represented by one to one orthology in each species. Therefore, two filtering steps were carried on to take away, first those genes for which an ortholog could not be found in one or more of the selected species, and second, those genes that had orthology one-to-many. Afterwards, selected species for this study were chosen in order to maximize the number of reported orthologs in all of them. For these highly informative sequences, we analyze adaptive selection at two distinct evolutionary time scales: the mammalian basal branch and in the human branch specifically.

### Mammalian high-throughput coding positive selection screening

As the number of sequences available for both background and foreground species is very high and could needlessly increase computational times, we first carried out an initial positive selection screening for a subset of mammalian and background genomes. We chose 14 default species including: human (*Homo sapiens*), olive baboon (*Papio anubis*), mouse (*Mus musculus*), guinea pig (*Cavia porcellus*), dog (*Canis familiaris*), cow (*Bos taurus*), pig (*Sus scrofa*), opossum (*Monodelphis domestica*), chicken (*Gallus gallus*), duck (*Anas platyrhynchos)*, collared flycatcher (*Ficedula albicollis*), turtle (*Pelodiscus sinensis*), anole lizard (*Anolis carolinensis*), and frog *(Xenopus tropicalis)*. If any of these species’ sequence was missing, the information was replaced by another equivalent coding sequence of a species belonging to the same taxon (see Supp. Information for additional info). We were careful to keep the representation of orders of our default species set.

To carry out this initial adaptive evolutionary analysis, we aligned all sequences using both PRANK aligner [139] and the OMM_MACSE framework, which includes aligning sequences with MAFFT aligner [140], cleaning these sequences with HMMCleaner [141] and applying MACSE to account for frameshifts and stop codons [142]. We used the intersection from these two methods to further explore these candidate genes in a similar fashion, increasing the total number of species (see methods section: mammalian extensive positive selection screening). Lastly, we used different approaches that aim to identify genes with evidence of positive selection at specific sites in the mammalian lineage: a codon-based positive selection analysis and an acceleration analysis. On the one hand, we used the modified Model A branch-site test 2 of positive selection [24] as applied in the *codeml* program from the PAML4 package [25] using the species tree phylogeny obtained from Ensembl v97. In this positive selection test, two-nested hypotheses were performed for the mammalian basal lineage as focal branch and their likelihoods were later compared: the alternative hypothesis (ω_0_ < 1, ω_1_ = 1, ω_2_ > 1) in which positive selection was allowed only in the selected focal branch and the null hypothesis where no positive selection was allowed (ω_0_ < 1, ω_1_ = 1). As ω_1_ is set to 1 representing null selection, in the alternative hypothesis two, ω values are calculated: ω_0_ and ω_2_ for the codons under negative and positive selection, respectively. In the null hypothesis, as no positively selected sites are allowed (no ω_2_), only one ω value must be estimated: ω_0_. The posterior probabilities of the given data to fit the proposed evolutionary model in each hypothesis were obtained by maximum likelihood optimization. A Likelihood Ratio Tests (LRTs) was performed from such probability values comparing twice the difference in log-likelihood probabilities for both hypotheses against a chi-square distribution with 1 degree of freedom. We split the optimization of the total number of parameters of the complex model of the positive selection test calculating all branch lengths before the branch-site positive selection analysis (see Supp. Information for additional information). We applied multiple testing corrections to the resulting *p* values through the Benjamini and Hochberg method or False Discovery Rate (FDR) in this high-throughput analysis and in the extensive screening [143]. Codon-based branch-site positive selection analyses allowed us to determine robust and strong signatures of adaptive evolution at the coding level.

Nevertheless, as a selective process at the nucleotide level might also be going on, the usage of acceleration analysis can be useful to detect evolutionary changes in coding conserved regions among vertebrates that have an increased substitution rate in the mammalian lineage. Therefore, we complemented our initial coding adaptive selection screening using coding accelerated elements derived from the TSARs database [26]. This approach allowed us to find signatures of accelerated evolution at the coding level for genes that either did not display signatures of positive selection through the PAML approach or could not be analyzed using this method due to missing sequence information or multiple orthology. In essence, we expanded the universe of genes analyzed in order to detect lineage-specific signatures of evolution.

In order to avoid local minimum solutions and improve the consistency of the branch-site positive selection analysis likelihood results among reruns of the same data, we opted for reducing these models’ parameter field dimensions. To do so, we split the optimization of the total number of parameters in two steps [144]. First, a basic model generally known in PAML as M0 or one-ratio model is run over the raw topology of the species tree to obtain the values of branch lengths through optimization by maximum likelihood under the Goldman & Yang codon-based evolutionary model of DNA evolution [145]. Once the branch lengths are estimated, the obtained phylogeny is assigned as the fixed input tree that will be used but not modified along the proper positive selection test. In this way, the branch lengths of the species tree, which comprises an important number of parameters to optimize, are pre-calculated in the M0 run, leaving only the model-specific parameters to be optimized during the proper positive selection test.

### Mammalian extensive positive selection screening

The candidate genes that were found to display positive selection in the batch analysis were re-analyzed using the maximum available number of sequences for each clade, up to 64. All of these multiple sequences were realigned using PRANK [139] and the OMM_MACSE framework [146] using MAFFT. Positively selected sites were identified through Bayes Empirical Bayes (posterior probability greater than 0.95) [147]. Even when, under both pipelines, the results are very similar, throughout the manuscript we report the set of genes derived from the OMM_MACSE pipeline, given that from very recent benchmarking assays it has been shown to most accurately represent positive selection [148].

### Human extensive positive selection screening

Sequences of up to 25 primates were aligned using PRANK [139] and the OMM_MACSE framework using MAFFT. We used the modified Model A branch-site test 2 of positive selection [24] in *codeml* to detect signatures of positive selection in the human branch. We report results based on the OMM_MACSE framework, but all analyses are provided in Supplementary Material and Tables. Additionally, selective sweeps were inspected using databases such as 1000 Genomes Selection Browser [24, 68], Ancient Positive Selection in Humans [70], and PopHumanScan [69] .

### Gene distribution and functions

Overrepresentation tests or functional enrichment analysis of gene ontology (G.O.) terms from molecular function, cellular component, and biological process were conducted with gProfiler (https://biit.cs.ut.ee/gprofiler/gost) using g:GOSt that maps genes to known functional information sources and detects statistically significantly enriched terms (version: e99_eg46_p14_f929183; April 2020) [149] using the following options: organisms: *Homo sapiens*, statistical domain scope: all known genes, and significance threshold: gSCS, 0.05. Protein interaction assessment was conducted using STRING (v11.0) with a combined medium confidence setting of 0.400 [150].

### Non-coding conserved regions analysis

Genomic coordinates for 4797 Therian Specific Accelerated Regions (TSARs) [26] and 2745 non-redundant Human Accelerated Regions (HARs) [71] were downloaded from the original publications as BED files. As the TSARs can be found in both coding and non-coding regions, elements that overlapped exons of the gene sets were filtered into noncoding TSARs (ncTSARs) and coding TSARs (cTSARs). The database of HARs that we used is composed only of noncoding regions [71]. Genomic coordinates for genes from our HLG database were obtained by subsetting gene names with UCSC Table Browser (GRCh37/hg19). Missing gene identifiers that were not automatically recognized by the UCSC Table Browser were manually inspected and replaced for its name synonyms. One BED record was created per whole gene using the transcriptional start site and the transcription end values from the knownCanonical table from the UCSC Genes track. This canonical transcript reports the longest coding sequence for each entry and includes all the non-coding introns. There were 11 unretrieved coordinates which belonged to mouse genes from the IMPC dataset that did not have an ortholog in the human genome. We evaluated how many of these accelerated elements (TSARs and HARs) overlapped non-exonic portions of the transcriptional units of hearing loss genes. Exons were obtained by exporting a BED file with UCSC Table Browser [UCSC Genes track: knownGene table/Exons].

To assign accelerated regions to intergenic regions, we took two approaches. First, we defined a gene regulatory domain, such as that applied in GREAT (http://great.stanford.edu/public/html/) [151] and other studies [152]. Here, each gene is assigned a basal regulatory domain of a minimum distance upstream and downstream of the TSS (regardless of other nearby genes). The gene regulatory domain is extended in both directions to the nearest gene’s basal domain but no more than the maximum extension (up to 1000 kb) in one direction. Second, we used chromatin conformation data from other tissues to define topologically associated domains (TADs), since this data is unfortunately inexistent for the inner ear. TADs are thought to be highly conserved among cell types [153–156] and possibly even among species [157]. We considered accelerated elements located in both intronic regions and non-coding exonic regions. Finally, we calculated the percentage of non-coding accelerated elements that could potentially interact with HL genes and compared these proportions to the percentage of HL genes with coding signals (calculated either via PAML and coding TSARs).

### Regulatory function testing of non-coding accelerated sequences

To test selected sequences as transcriptional enhancers, we used an enhancer assay in transgenic zebrafish. Genomic regions containing each selected TSAR were amplified by proofreading PCR from human samples and cloned individually in the vector pXIG_cFos containing the *cfos* minimal promoter and the reporter gene *EGFP* that was kindly donated by Andrew McCallion (Additional file 1: Table S9). Transgenic zebrafish were produced as previously described [158, 159]. Each TSAR-cFos construct was co-injected with transposase mRNA in one-cell zebrafish embryos. Injected embryos were raised until adulthood and F1 were obtained by natural mating with WT animals. At least 3 stable transgenic lines were obtained per construct, except for EXOC4-TSAR.2949 (2 lines) and EXOC4-TSAR.1392 (no stable lines). Microscopy was carried out on tricaine-anesthetized embryos mounted in 3% methylcellulose. Neuromasts were stained using a working solution of 1.4 μM FM® 4-64 (ThermoFisher). Zebrafish were immersed in this solution for 2 min, with very gentle stirring in a Petri dish. The solution was then rinsed and animals were photographed immediately. Whole-mount images were taken on an Olympus BX41 fluorescence microscope with an Olympus DP71 digital camera. Confocal images were obtained with a Leica SPE microscope. Zebrafish experiments were performed in wild-type AB lines [160], in accordance with approved protocols by the Institutional Animal Studies Committee.

## Supplementary Information

**Additional file 1: Supp. Tables S1 to S10.** Table S1. Initial screening of hearing loss genes (*n* = 420) for signatures of coding positive selection in the mammalian basal branch. Table S2. Extensive analysis of 58 candidate positively selected hearing loss genes obtained from initial screening. Table S3. Screening of coding positive selection in the human branch, using a background of primate species for our hearing loss database (*n* = 420). Table S4. Screening of non-coding acceleration in the mammalian and human branches. Table S5. Functional evidence of regulatory activity in non-coding accelerated elements in the mammalian and human branches. Table S6. Effect of variation in positively selected sites using human protein as reference. Table S7. Gene ontology analyses for mammal coding positive selected genes against the hearing loss database. Table S8. Gene ontology analyses for different sets of genes: non-coding elements in mammals (ncTSARs) vs. coding positive selection in mammals. Table S9. Information for candidate regulatory regions in hearing loss genes tested in enhancer assays in transgenic zebrafish. Table S10. Summary of distinct approaches performed to test the relative contribution of coding and non-coding elements for each hearing loss gene.

**Additional file 2: Supp. Figures S1-S13.** FigS1. Phylogenetic tree and positive selected sites of an essential tip link protein: CDH23. Fig S2. Phylogenetic tree and positive selected sites of the key inner hair cell gene OTOF. Fig S3. Phylogenetic tree and positive selected sites of the hair cell gene LOXHD1. Fig S4. Enhancer assays in stable transgenic zebrafish for the DIAPH3-TSAR.1094 noncoding element. Fig S5. Enhancer assays for the *EXOC4* accelerated elements in transgenic zebrafish. Fig S6. Enhancer assays in stable transgenic zebrafish for the SMOC1-TSAR3685 noncoding element. Fig S7. Enhancer assays in stable transgenic zebrafish for the MIPOL1-TSAR.2840 noncoding element. Fig S8. Enhancer assays in stable transgenic zebrafish for the GATA2-TSAR.3936 noncoding element. Fig S9. Comparative enhancer assays in transgenic zebrafish of the accelerated sequence JAZF1-TSAR.4204. Fig S10. JAZF1-TSAR.4204-Hs directs the expression to neuromast in the developing zebrafish. Fig S11. Comparative analysis of genes under coding positive selection vs. non-coding acceleration in mammals. Fig S12. Comparative analysis of genes with signatures of non-coding acceleration in mammals vs. non-coding acceleration in humans. Fig S13. Analysis of overlap among different sets of genes and network association analyses.

**Additional file 3: Supp. Table S11.** Table S11. Expression analysis of enhancer assays in transgenic zebrafish

## Data Availability

All data generated or analyzed during this study are included in this published article [and its supplementary information files]. Alignment files and code used for the analysis were deposited in figshare: https://figshare.com/s/afde8f3c7bc03f15413c [161].
