# Supplementary material for: Hearing loss genes reveal patterns of adaptive evolution at the coding and non-coding levels in mammals
Source: BMC Biol. 2021 Nov 16;19:244. doi: 10.1186/s12915-021-01170-6 (PMC8594068; doi:10.1186/s12915-021-01170-6)
Supplement: Supplementary file 2 — Additional file 2: Supp. Figures S1-S13. FigS1. Phylogenetic tree and positive selected sites of an essential tip link protein: CDH23. Fig S2. Phylogenetic tree and positive selected sites of the key inner hair cell gene OTOF. Fig S3. Phylogenetic tree and positive selected sites of the hair cell gene LOXHD1. Fig S4. Enhancer assays in stable transgenic zebrafish for the DIAPH3-TSAR.1094 noncoding element. Fig S5. Enhancer assays for the EXOC4 accelerated elements in transgenic zebrafish. Fig S6. Enhancer assays in stable transgenic zebrafish for the SMOC1-TSAR3685 noncoding element. Fig S7. Enhancer assays in stable transgenic zebrafish for the MIPOL1-TSAR.2840 noncoding element. Fig S8. Enhancer assays in stable transgenic zebrafish for the GATA2-TSAR.3936 noncoding element. Fig S9. Comparative enhancer assays in transgenic zebrafish of the accelerated sequence JAZF1-TSAR.4204. Fig S10. JAZF1-TSAR.4204-Hs directs the expression to neuromast in the developing zebrafish. Fig S11. Comparative analysis of genes under coding positive selection vs. non-coding acceleration in mammals. Fig S12. Comparative analysis of genes with signatures of non-coding acceleration in mammals vs. non-coding acceleration in humans. Fig S13. Analysis of overlap among different sets of genes and network association analyses. [file 12915_2021_1170_MOESM2_ESM.pdf]

Supplementary Figure S1

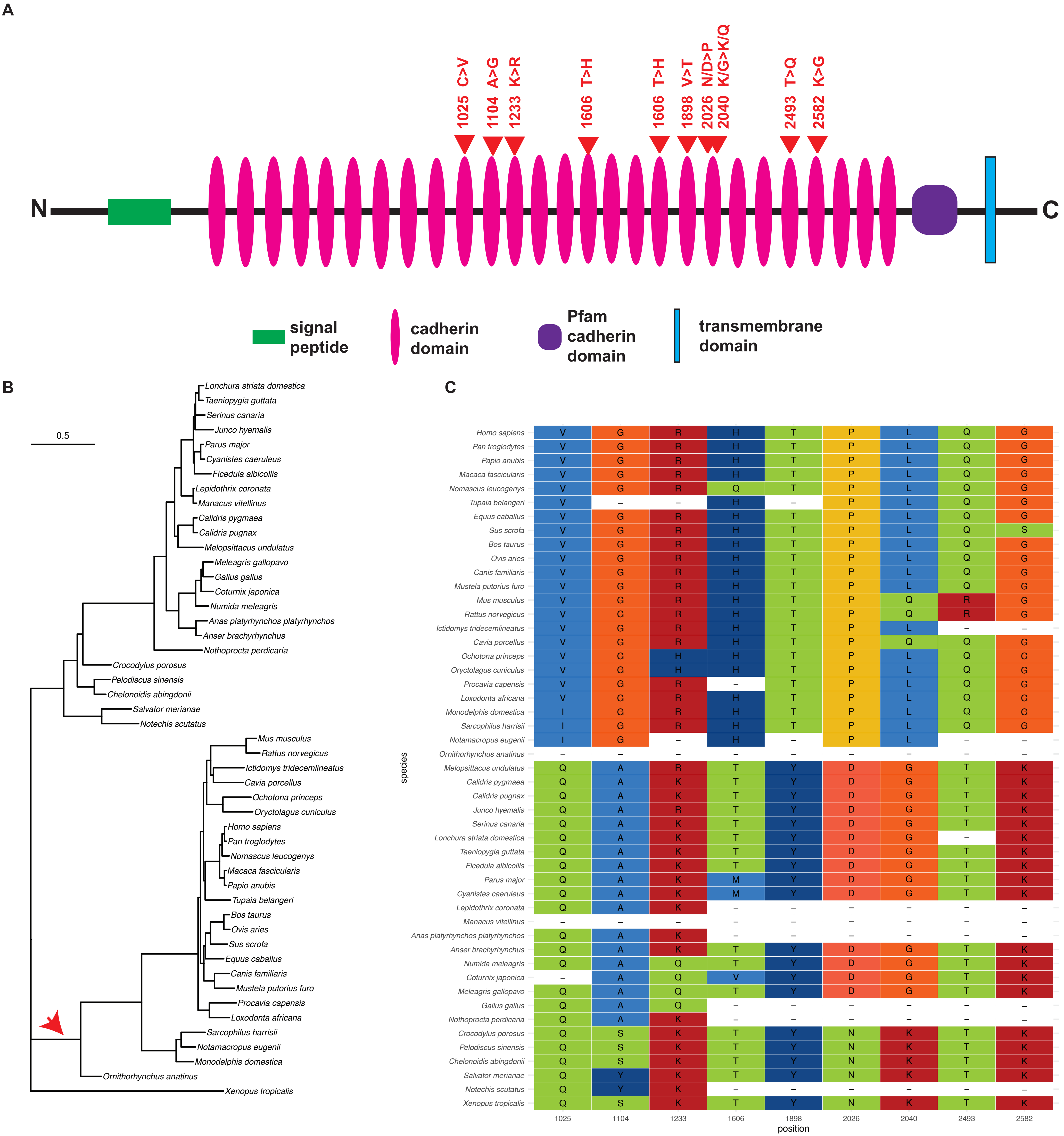

Supplementary Figure S2

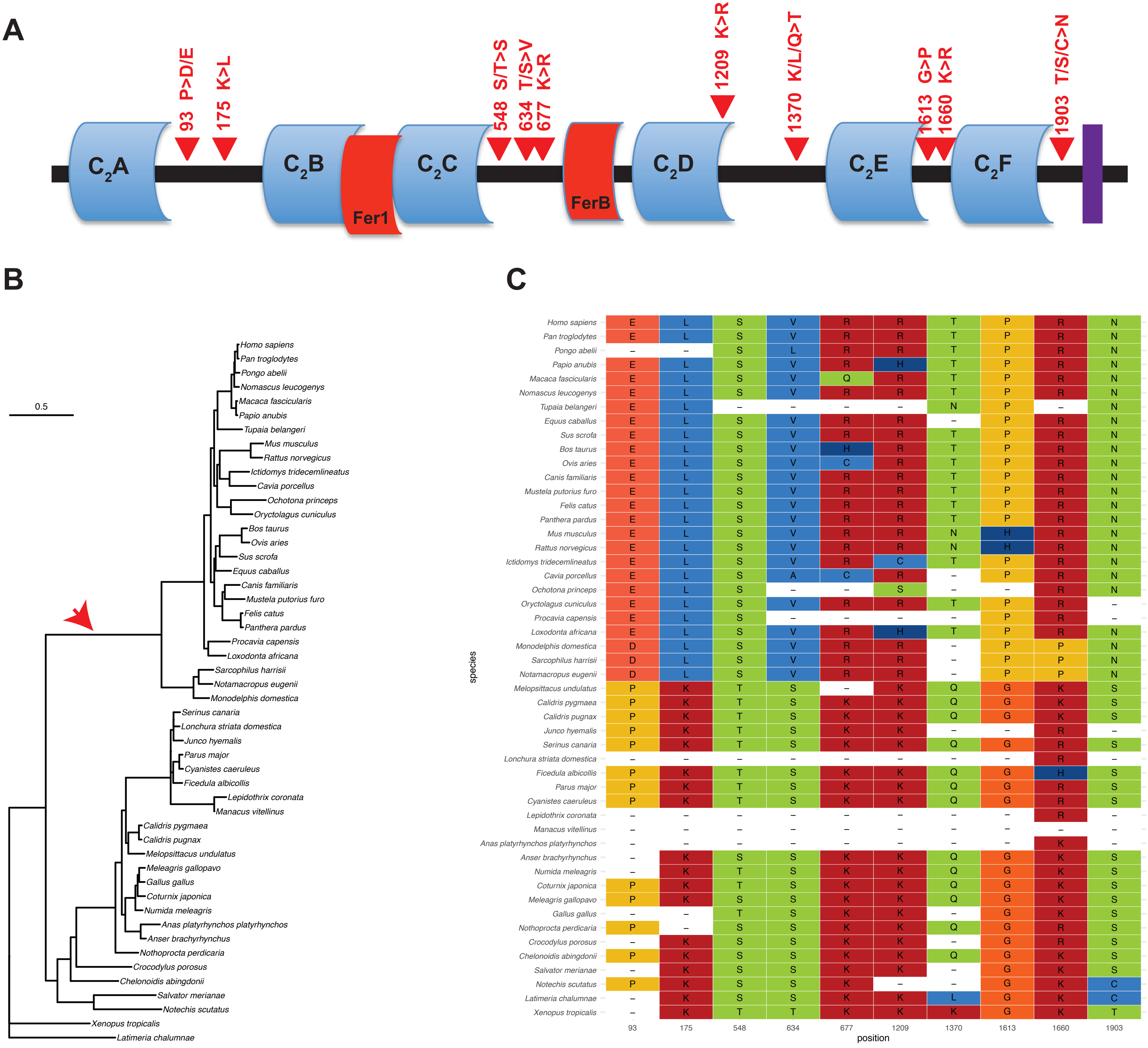

Supplementary Figure S3

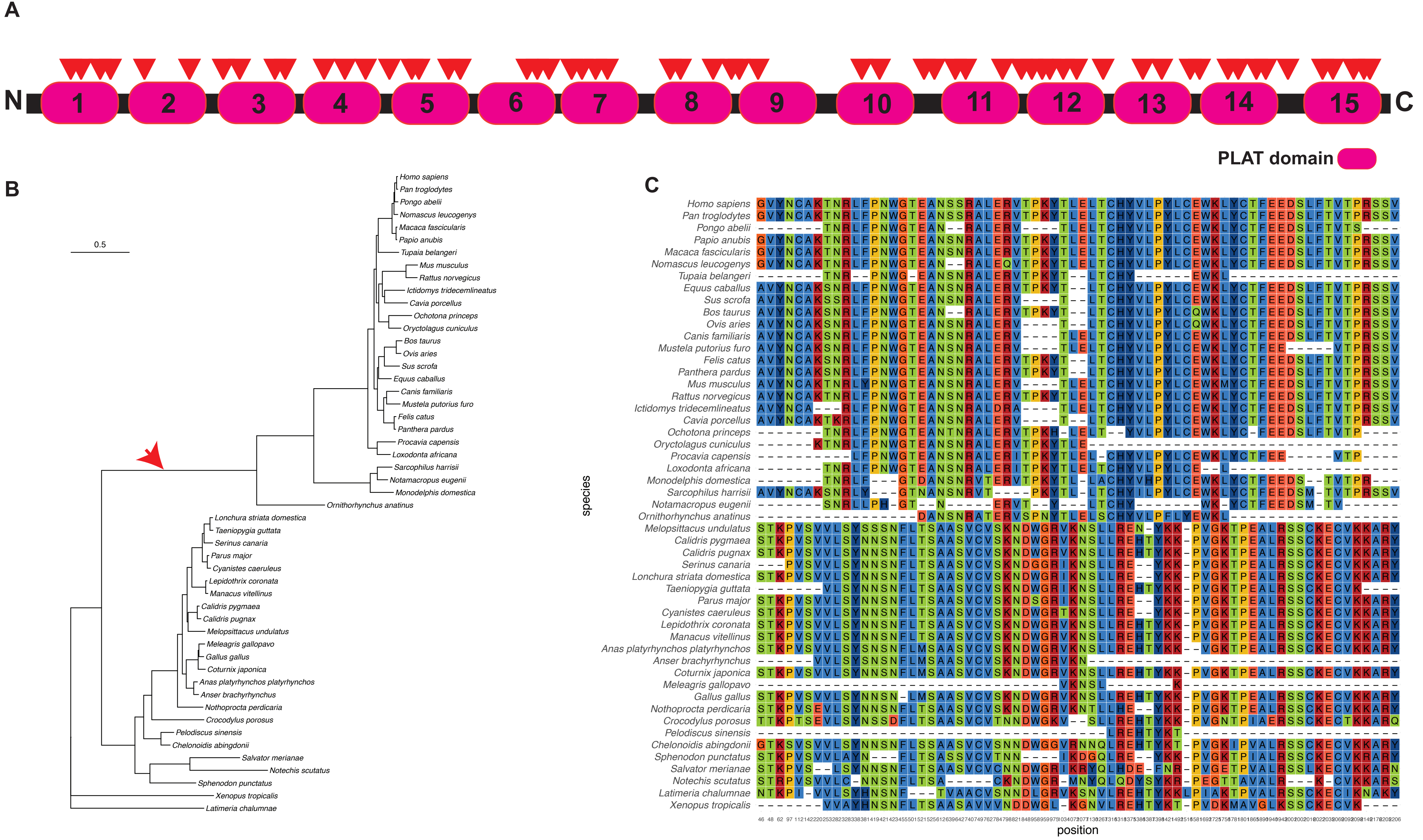

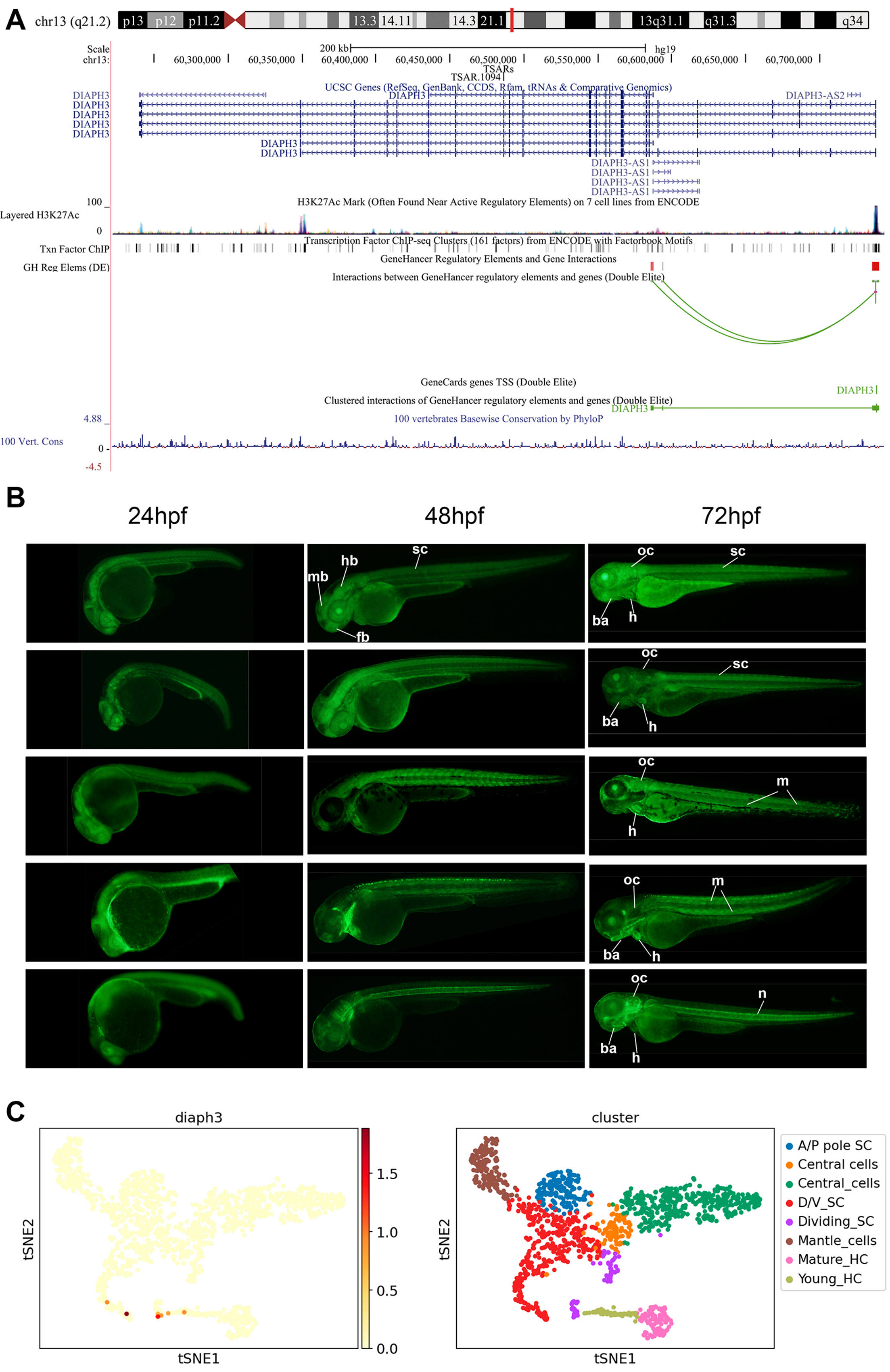

10.7554/eLife.44431.

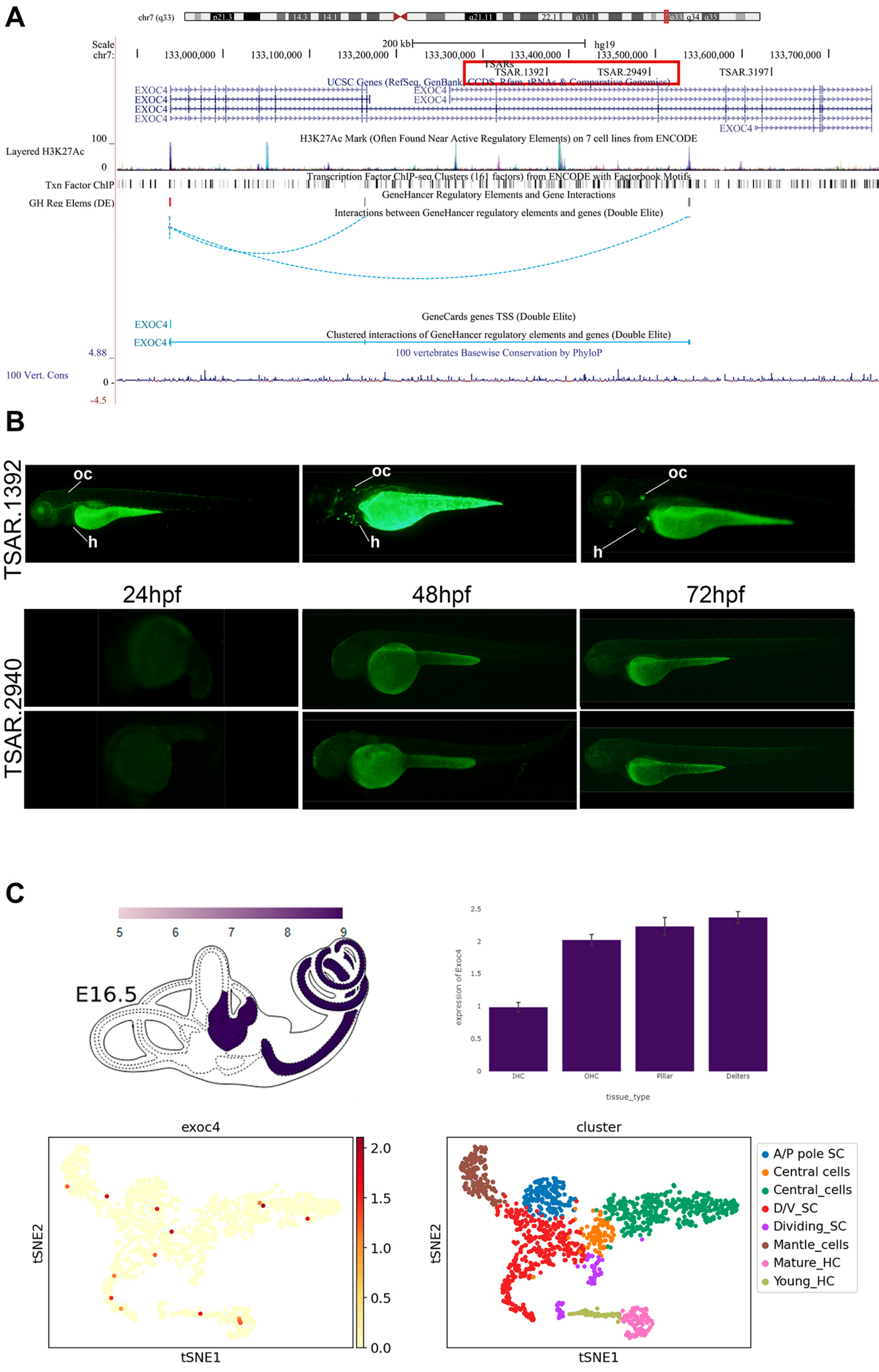

A
chr7 (q33)
Scale
chr7:
200 kb
hg19
133,000,000
133,100,000
133,200,000
133,300,000
133,400,000
133,500,000
133,600,000
133,700,000
TSARs
TSAR.1392
TSAR.2949
TSAR.3197
UCSC Genes (RefSeq, GenBank, CCDS, Rfam, tRNAs & Comparative Genomics)
EXOC4
Layered H3K27Ac
H3K27Ac Mark (Often Found Near Active Regulatory Elements) on 7 cell lines from ENCODE
Txn Factor ChIP
Transcription Factor ChIP-seq Clusters (161 factors) from ENCODE with Factorbook Motifs
GH Reg Elems (DE)
GeneHancer Regulatory Elements and Gene Interactions
Interactions between GeneHancer regulatory elements and genes (Double Elite)
GeneCards genes TSS (Double Elite)
Clustered interactions of GeneHancer regulatory elements and genes (Double Elite)
100 vertebrates Basewise Conservation by PhyloP
100 Vert. Cons
B
TSAR.1392
oc
h
24hpf
48hpf
72hpf
TSAR.2940
C
E16.5
5
6
7
8
9
expression of Exoc4
IHC
OHC
Pillar
Deiters
tissue_type
exoc4
tSNE1
tSNE2
cluster
A/P pole SC
Central cells
Central_cells
D/V_SC
Dividing_SC
Mantle_cells
Mature_HC
Young_HC

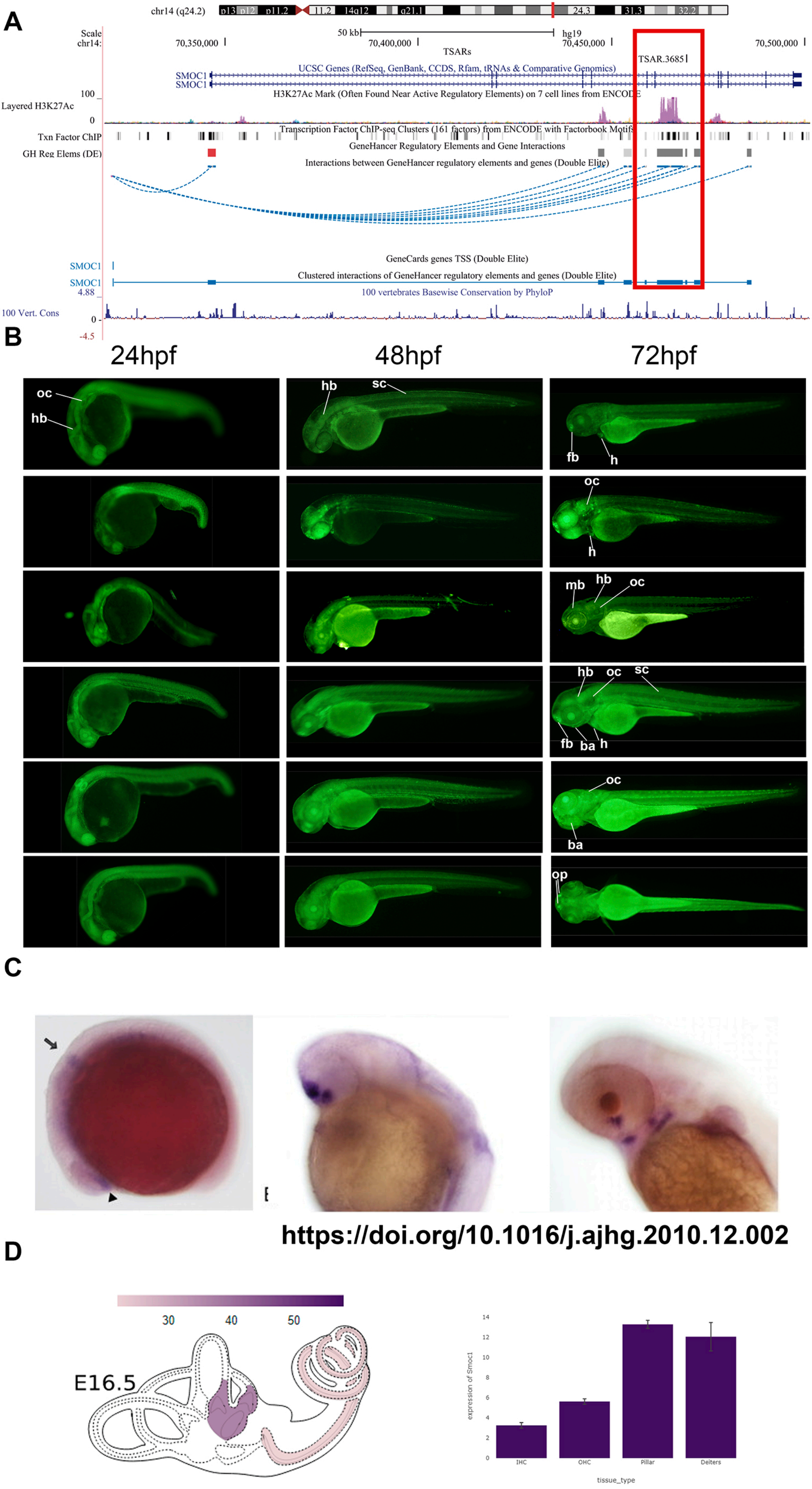
A
chr14 (q24.2)
Scale
chr14:
50 kb
hg19
70,350,000
70,400,000
70,450,000
70,500,000
TSARs
TSAR.3685
UCSC Genes (RefSeq, GenBank, CCDS, Rfam, tRNAs & Comparative Genomics)
SMOC1
SMOC1
100
Layered H3K27Ac
0
H3K27Ac Mark (Often Found Near Active Regulatory Elements) on 7 cell lines from ENCODE
Txn Factor ChIP
Transcription Factor ChIP-seq Clusters (161 factors) from ENCODE with Factorbook Motifs
GH Reg Elems (DE)
GeneHancer Regulatory Elements and Gene Interactions
Interactions between GeneHancer regulatory elements and genes (Double Elite)
GeneCards genes TSS (Double Elite)
SMOC1
Clustered interactions of GeneHancer regulatory elements and genes (Double Elite)
SMOC1
4.88
100 vertebrates Basewise Conservation by PhyloP
100 Vert. Cons
0
-4.5
B
24hpf
48hpf
72hpf
oc
hb
sc
fb
h
mb
ba
op
C
https://doi.org/10.1016/j.ajhg.2010.12.002
D
30
40
50
E16.5
expression of Smoc1
tissue_type
IHC
OHC
Pillar
Deiters
0
2
4
6
8
10
12
14

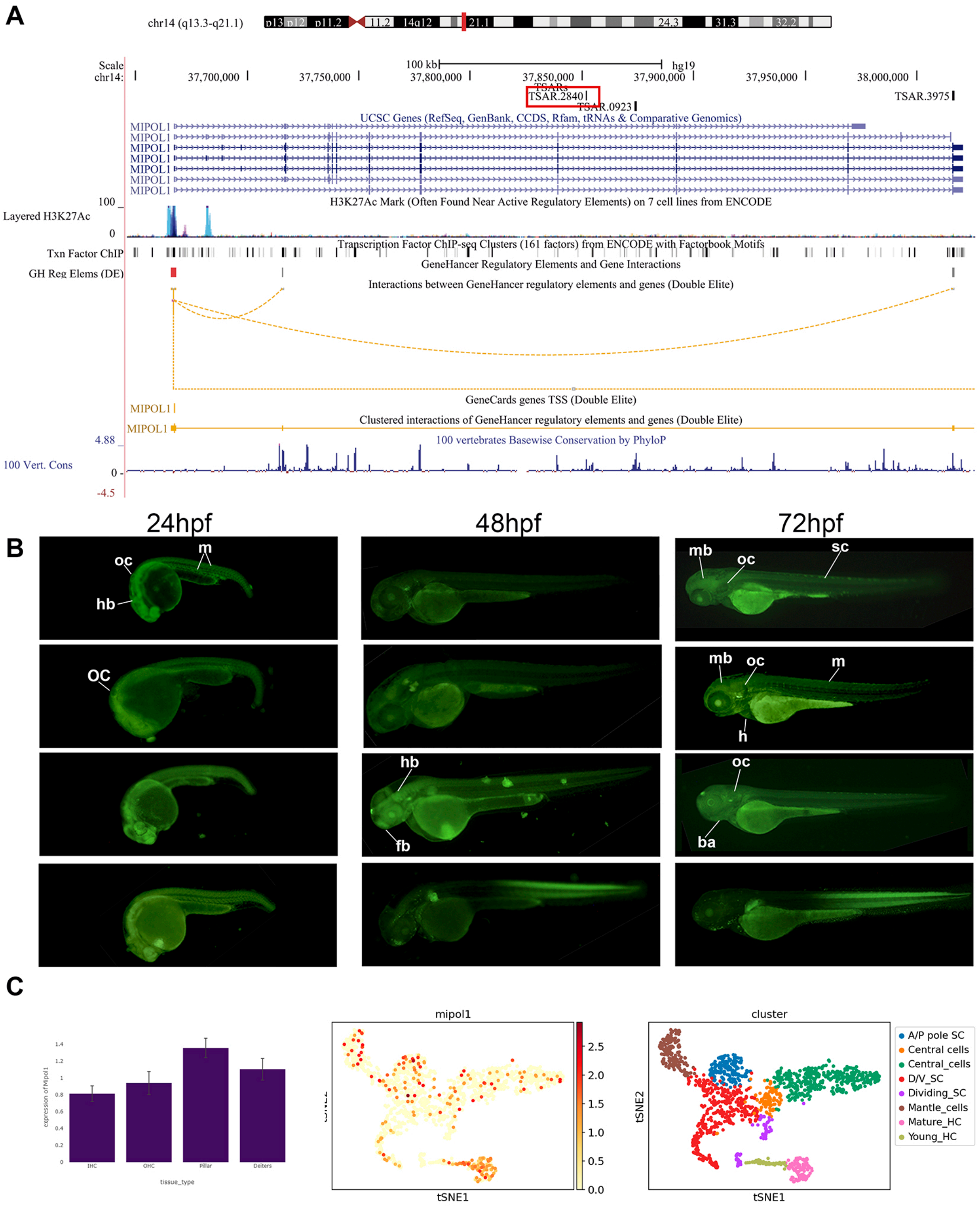
A
chr14 (q13.3-q21.1)
Scale
chr14:
100 kb
hg19
37,700,000
37,750,000
37,800,000
37,850,000
37,900,000
37,950,000
38,000,000
TSARs
TSAR.2840
TSAR.0923
TSAR.3975
UCSC Genes (RefSeq, GenBank, CCDS, Rfam, tRNAs & Comparative Genomics)
MIPOL1
Layered H3K27Ac
H3K27Ac Mark (Often Found Near Active Regulatory Elements) on 7 cell lines from ENCODE
Txn Factor ChIP
Transcription Factor ChIP-seq Clusters (161 factors) from ENCODE with Factorbook Motifs
GH Reg Elems (DE)
GeneHancer Regulatory Elements and Gene Interactions
Interactions between GeneHancer regulatory elements and genes (Double Elite)
GeneCards genes TSS (Double Elite)
Clustered interactions of GeneHancer regulatory elements and genes (Double Elite)
100 Vert. Cons
100 vertebrates Basewise Conservation by PhyloP
B
24hpf
48hpf
72hpf
oc
m
hb
mb
sc
h
fb
ba
C
expression of Mipol1
tissue_type
IHC
OHC
Pillar
Deiters
mipol1
cluster
tSNE1
tSNE2
A/P pole SC
Central cells
Central_cells
D/V_SC
Dividing_SC
Mantle_cells
Mature_HC
Young_HC

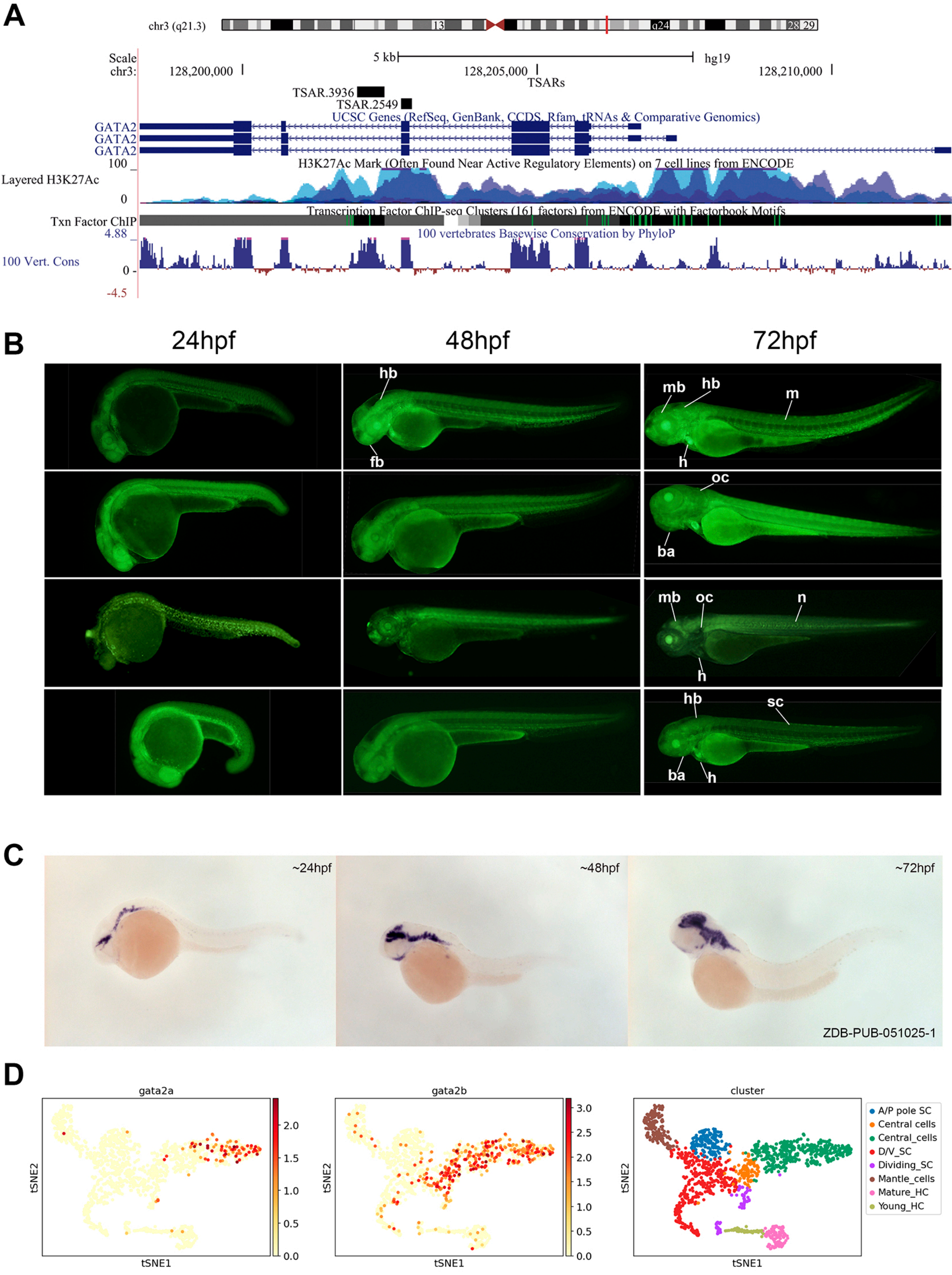
A
chr3 (q21.3)
13
q24
28 29
Scale
chr3:
5 kb
hg19
128,200,000
128,205,000
128,210,000
TSARs
TSAR.3936
TSAR.2549
UCSC Genes (RefSeq, GenBank, CCDS, Rfam, tRNAs & Comparative Genomics)
GATA2
GATA2
GATA2
100
Layered H3K27Ac
0
H3K27Ac Mark (Often Found Near Active Regulatory Elements) on 7 cell lines from ENCODE
Txn Factor ChIP
Transcription Factor ChIP-seq Clusters (161 factors) from ENCODE with Factorbook Motifs
4.88
100 vertebrates Basewise Conservation by PhyloP
100 Vert. Cons
0
-4.5
B
24hpf
48hpf
72hpf
hb
fb
mb
hb
m
h
oc
ba
mb
oc
n
h
hb
sc
ba
h
C
~24hpf
~48hpf
~72hpf
ZDB-PUB-051025-1
D
gata2a
gata2b
cluster
tSNE2
tSNE1
A/P pole SC
Central cells
Central_cells
D/V_SC
Dividing_SC
Mantle_cells
Mature_HC
Young_HC

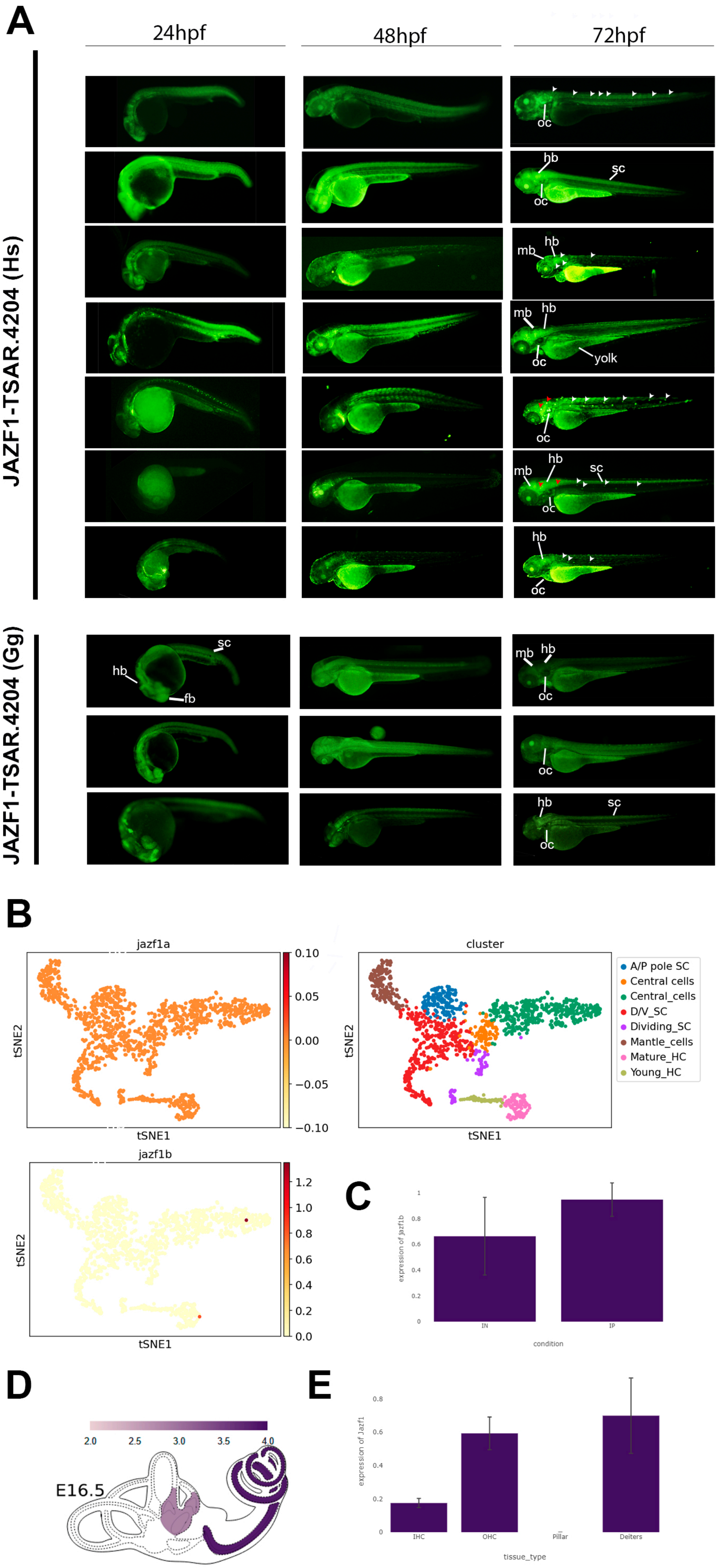

A
24hpf
48hpf
72hpf
JAZF1-TSAR.4204 (Hs)
JAZF1-TSAR.4204 (Gg)
oc
hb
sc
mb
yolk
fb
B
jazf1a
cluster
tSNE1
tSNE2
A/P pole SC
Central cells
Central_cells
D/V_SC
Dividing_SC
Mantle_cells
Mature_HC
Young_HC
jazf1b
C
expression of jazf1b
IN
IP
condition
D
E16.5
E
expression of Jazf1
IHC
OHC
Pillar
Deiters
tissue_type

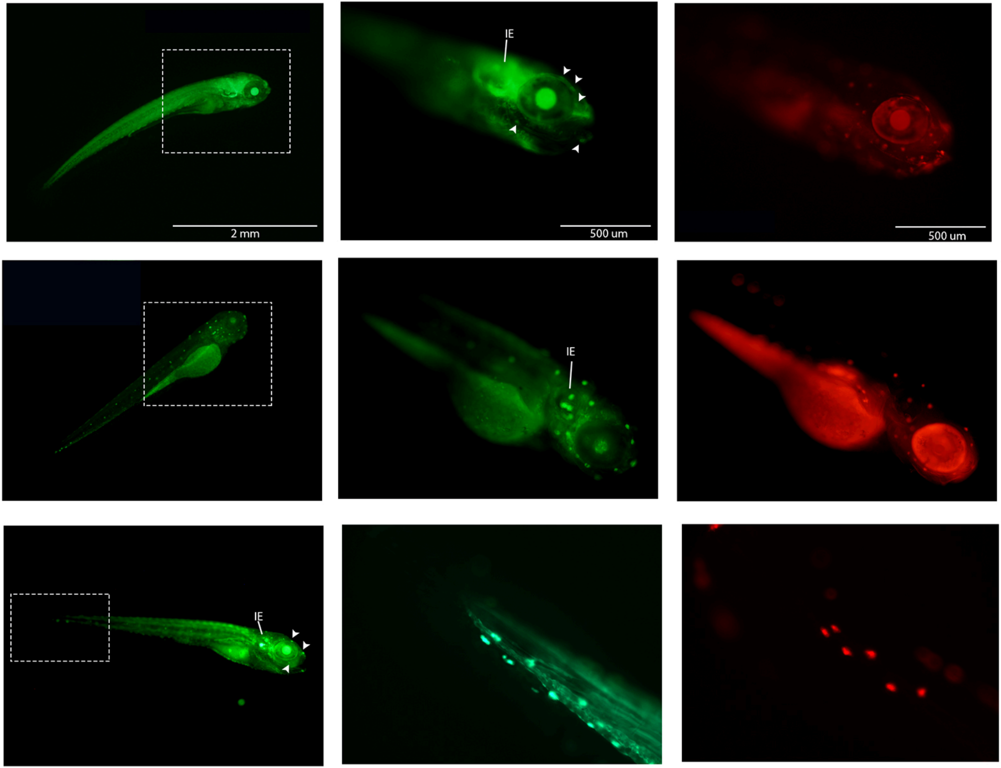

2 mm
IE
500 um
500 um
IE
IE

Supplementary Figure S11

GO:MF				PAML mammals			ncTSARs		
Term Name	Term ID	T	U	p_adj	Q	Q∩T	p_adj	Q	Q∩T
molecular_function	GO:0003674	18130	60624	3.825×10^{-19}	53	49	9.871×10^{-6}	14	14
binding	GO:0005488	15960	60624	1.732×10^{-16}	53	45	1.656×10^{-6}	14	14
protein binding	GO:0005515	12784	60624	1.068×10^{-13}	53	39	3.955×10^{-6}	14	13
calmodulin binding	GO:0005516	200	60624	1.380×10^{-7}	53	7	1.000		
cytoskeletal protein binding	GO:0008092	990	60624	2.262×10^{-7}	53	11	1.000	14	2
actin binding	GO:0003779	432	60624	2.747×10^{-5}	53	7	9.429×10^{-1}	14	2
ion binding	GO:0043167	6310	60624	3.817×10^{-5}	53	20	3.930×10^{-1}	14	6
actin filament binding	GO:0051015	200	60624	2.494×10^{-4}	53	5	1.000	14	1
actin-dependent ATPase activity	GO:0030898	24	60624	3.352×10^{-4}	53	3	1.000		
protein-containing complex binding	GO:0044877	1249	60624	3.394×10^{-4}	53	9	1.000	14	1
Ras GTPase binding	GO:0017016	389	60624	1.000	53	3	3.437×10^{-4}	14	4
small GTPase binding	GO:0031267	410	60624	1.000	53	3	4.234×10^{-4}	14	4
microfilament motor activity	GO:0000146	29	60624	6.033×10^{-4}	53	3	1.000		
GTPase binding	GO:0051020	547	60624	1.000	53	3	1.322×10^{-3}	14	4
cis-regulatory region sequence-specific DNA binding	GO:0000987	633	60624	1.000	53	1	2.347×10^{-3}	14	4
carbohydrate derivative binding	GO:0097367	2270	60624	6.317×10^{-3}	53	10	1.000	14	1
transcription regulator activity	GO:0140110	1718	60624	1.000	53	2	6.359×10^{-3}	14	5
calcium transmembrane transporter activity, phosphory...	GO:0005388	9	60624	7.152×10^{-3}	53	2	1.000		
anion binding	GO:0043168	2847	60624	7.833×10^{-3}	53	11	1.000	14	1
transcription regulatory region sequence-specific DNA ...	GO:0000976	888	60624	1.000	53	1	8.813×10^{-3}	14	4
regulatory region nucleic acid binding	GO:0001067	889	60624	1.000	53	1	8.852×10^{-3}	14	4
calcium ion binding	GO:0005509	717	60624	1.020×10^{-2}	53	6	1.000	14	1
sequence-specific double-stranded DNA binding	GO:1990837	930	60624	1.000	53	1	1.055×10^{-2}	14	4
nuclear receptor activity	GO:0004879	47	60624	1.000	53	1	1.152×10^{-2}	14	2
ligand-activated transcription factor activity	GO:0098531	47	60624	1.000	53	1	1.152×10^{-2}	14	2
metal ion transmembrane transporter activity	GO:0046873	442	60624	1.152×10^{-2}	53	5	1.000		
metal ion binding	GO:0046872	4222	60624	1.414×10^{-2}	53	13	4.155×10^{-1}	14	5
DNA-binding transcription factor activity, RNA polymer...	GO:0000981	1021	60624	1.000	53	1	1.515×10^{-2}	14	4
double-stranded DNA binding	GO:0003690	1022	60624	1.000	53	2	1.520×10^{-2}	14	4
steroid hormone receptor activity	GO:0003707	56	60624	1.000	53	1	1.639×10^{-2}	14	2
PDZ domain binding	GO:0030165	87	60624	1.688×10^{-2}	53	3	1.000	14	1
cation binding	GO:0043169	4316	60624	1.773×10^{-2}	53	13	4.582×10^{-1}	14	5
inorganic molecular entity transmembrane transporter a...	GO:0015318	828	60624	2.254×10^{-2}	53	6	1.000		
enzyme binding	GO:0019899	2242	60624	1.848×10^{-1}	53	8	2.256×10^{-2}	14	5
nucleotide binding	GO:0000166	2164	60624	2.637×10^{-2}	53	9	1.000	14	1
nucleoside phosphate binding	GO:1901265	2165	60624	2.646×10^{-2}	53	9	1.000	14	1
cell adhesion molecule binding	GO:0050839	535	60624	2.828×10^{-2}	53	5	1.000	14	1
sequence-specific DNA binding	GO:0043565	1221	60624	1.000	53	1	3.019×10^{-2}	14	4
ion transmembrane transporter activity	GO:0015075	879	60624	3.122×10^{-2}	53	6	1.000		
DNA binding	GO:0003677	2485	60624	1.000	53	4	3.660×10^{-2}	14	5
DNA-binding transcription factor activity	GO:0003700	1300	60624	1.000	53	1	3.840×10^{-2}	14	4
heterocyclic compound binding	GO:1901363	6270	60624	5.882×10^{-2}	53	15	4.846×10^{-2}	14	7

Supplementary Figure S12

GO:MF				ncTSARs			HARs		
Term Name	Term ID	T	U	p_adj	Q	$Q \cap T$	p_adj	Q	$Q \cap T$
molecular_function	GO:0003674	18130	60624	9.871×10^{-6}	14	14	1.185×10^{-10}	23	23
binding	GO:0005488	15960	60624	1.656×10^{-6}	14	14	4.124×10^{-10}	23	22
protein binding	GO:0005515	12784	60624	3.955×10^{-6}	14	13	1.019×10^{-6}	23	18
ion binding	GO:0043167	6310	60624	3.930×10^{-1}	14	6	9.794×10^{-5}	23	12
Ras GTPase binding	GO:0017016	389	60624	3.437×10^{-4}	14	4	1.000	23	2
protein kinase activity	GO:0004672	615	60624	1.000	14	1	4.177×10^{-4}	23	5
small GTPase binding	GO:0031267	410	60624	4.234×10^{-4}	14	4	1.000	23	2
transmembrane receptor protein kinase activity	GO:0019199	82	60624	1.000			5.658×10^{-4}	23	3
heterocyclic compound binding	GO:1901363	6270	60624	4.846×10^{-2}	14	7	8.090×10^{-4}	23	11
phosphotransferase activity, alcohol group as acceptor	GO:0016773	720	60624	1.000	14	1	8.972×10^{-4}	23	5
organic cyclic compound binding	GO:0097159	6355	60624	5.275×10^{-2}	14	7	9.225×10^{-4}	23	11
GTPase binding	GO:0051020	547	60624	1.322×10^{-3}	14	4	1.545×10^{-1}	23	3
kinase activity	GO:0016301	824	60624	1.000	14	1	1.719×10^{-3}	23	5
metal ion binding	GO:0046872	4222	60624	4.155×10^{-1}	14	5	1.735×10^{-3}	23	9
catalytic activity, acting on a protein	GO:0140096	2221	60624	1.000	14	2	1.752×10^{-3}	23	7
cation binding	GO:0043169	4316	60624	4.582×10^{-1}	14	5	2.072×10^{-3}	23	9
cis-regulatory region sequence-specific DNA binding	GO:0000987	633	60624	2.347×10^{-3}	14	4	1.000		
transferase activity, transferring phosphorus-containing...	GO:0016772	1003	60624	1.000	14	1	4.404×10^{-3}	23	5
transcription regulator activity	GO:0140110	1718	60624	6.359×10^{-3}	14	5	1.000	23	3
transcription regulatory region sequence-specific DNA ...	GO:0000976	888	60624	8.813×10^{-3}	14	4	1.000	23	1
regulatory region nucleic acid binding	GO:0001067	889	60624	8.852×10^{-3}	14	4	1.000	23	1
sequence-specific double-stranded DNA binding	GO:1990837	930	60624	1.055×10^{-2}	14	4	1.000	23	1
ligand-activated transcription factor activity	GO:0098531	47	60624	1.152×10^{-2}	14	2	1.000		
nuclear receptor activity	GO:0004879	47	60624	1.152×10^{-2}	14	2	1.000		
DNA-binding transcription factor activity, RNA polymer...	GO:0000981	1021	60624	1.515×10^{-2}	14	4	1.000	23	2
double-stranded DNA binding	GO:0003690	1022	60624	1.520×10^{-2}	14	4	1.000	23	2
steroid hormone receptor activity	GO:0003707	56	60624	1.639×10^{-2}	14	2	1.000		
carbohydrate derivative binding	GO:0097367	2270	60624	1.000	14	1	2.179×10^{-2}	23	6
enzyme binding	GO:0019899	2242	60624	2.256×10^{-2}	14	5	1.000	23	4
ATP binding	GO:0005524	1499	60624	1.000	14	1	2.911×10^{-2}	23	5
sequence-specific DNA binding	GO:0043565	1221	60624	3.019×10^{-2}	14	4	1.000	23	1
adenyl ribonucleotide binding	GO:0032559	1557	60624	1.000	14	1	3.470×10^{-2}	23	5
cadherin binding	GO:0045296	329	60624	1.000	14	1	3.535×10^{-2}	23	3
adenyl nucleotide binding	GO:0030554	1568	60624	1.000	14	1	3.584×10^{-2}	23	5
DNA binding	GO:0003677	2485	60624	3.660×10^{-2}	14	5	2.854×10^{-1}	23	5
transmembrane receptor protein tyrosine kinase activity	GO:0004714	64	60624	1.000			3.738×10^{-2}	23	2
DNA-binding transcription factor activity	GO:0003700	1300	60624	3.840×10^{-2}	14	4	1.000	23	2

Supplementary Figure S13

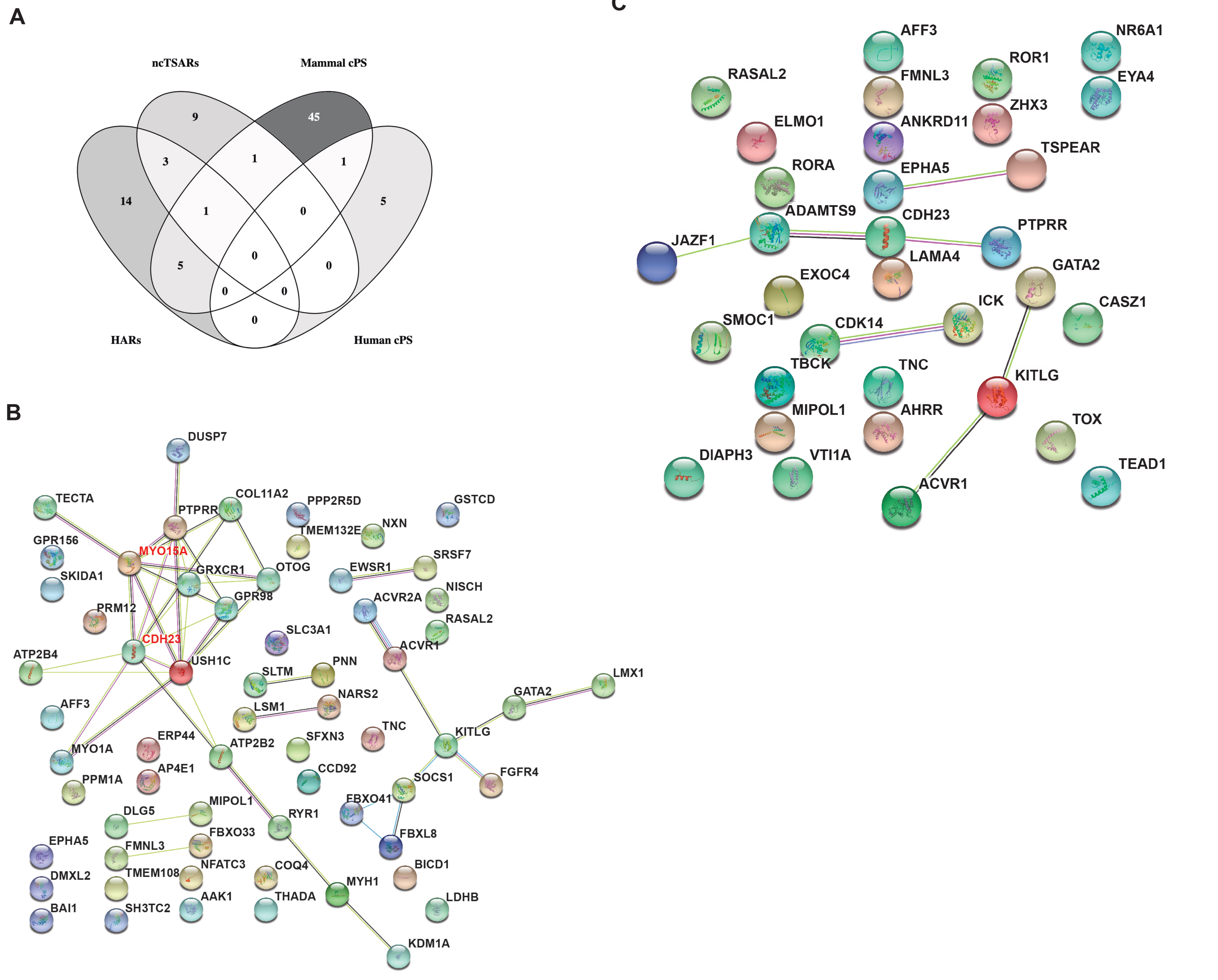

Supplementary Figure Legends

Supplementary Figure S1. Phylogenetic tree and positive selected sites of an essential tip link protein: CDH23.
(A) Schematic diagram of the CDH23 protein domains showing the approximate localization of positively selected sites (red arrows). Protein domains were approximately depicted according to [1]. (B) Phylogenetic tree showing vertebrate species used for the analysis. The red arrow indicates the foreground branch. (C) Sequence alignment of the positively selected sites identified. Mammalian species included in the foreground branch are in red. Positions of the human sequence given correspond to the sequence ENSP00000224721.

Supplementary Figure S2. Phylogenetic tree and positive selected sites of the key inner hair cell gene *OTOF*.
(A) Schematic diagram of the OTOF protein domains showing the approximate localization of positively selected sites (red arrows). Protein domains were approximately depicted according to [2]. (B) Phylogenetic tree showing vertebrate species used for the analysis. The red arrow indicates the foreground branch. (C) Sequence alignment of the positively selected sites identified. Mammalian species included in the foreground branch are in red. Positions of the human sequence given correspond to the sequence ENSP00000272371.

Supplementary Figure S3. Phylogenetic tree and positive selected sites of the hair cell gene *LOXHD1*.
(A) Schematic diagram of the LOXHD1 protein domains showing the approximate localization of positively selected sites (red arrows). Protein domains were approximately depicted according to [3].(B) Phylogenetic tree showing vertebrate species used for the analysis. The red arrow indicates the foreground branch. (C) Sequence alignment of the positively selected sites identified. Mammalian species included in the foreground branch are in red. Positions of the human sequence given correspond to the sequence ENSP00000444586

Supplementary Figure S4. Enhancer assays in stable transgenic zebrafish for the DIAPH3-TSAR.1094 noncoding element.
(A) Panel showing the genome locus of the gene *DIAPH3* in the human genome and the location of the TSAR elements. In the lower part of the panel GeneHancer and Conservation Tracks are displayed. Some regulatory elements in the *DIAPH3* locus show interaction with the *DIAPH3* promoter. (B) Fluorescent microphotographs of five whole mount stable transgenic zebrafish lines showing the expression pattern of the reporter gene eGFP driven by the DIAPH3-TSAR.1094 element. We show the best representative image for each line. (C) On the left panel, expression data for scRNA-seq on FACS sorted cells from the neuromast of 5dpf zebrafish embryos. The expression of diaph3 positive cells is depicted on the central diagram and the identified neuromast cell types are depicted on the right diagram [4]. Expression diagrams were obtained from

the gEAR portal (www.umgear.org) and scRNAseq plots were generated from (https://piotrowskilab.shinyapps.io/neuromast_homeostasis_scrnaseq_2018). Anatomic references: hb: hindbrain; mb: midbrain; hb: hindbrain; sc: spinal cord; oc: otic capsule; h: heart; ba: branchial arches; op: olfactory pits.

Supplementary Figure S5. Enhancer assays for the EXOC4 accelerated elements in transgenic zebrafish.
(A) Panel showing the genome locus of the gene *EXOC4* in the human genome and the location of the TSAR elements. In addition, the figure shows supported interactions between the noncoding elements and the *EXOC4* promoter predicted by GeneHancer track in UCSC Genome Browser. Some regulatory elements in the *EXOC4* have evidence of interaction with the *EXOC4* promoter. (B) Fluorescent microphotographs of three representative whole mount transient (EXOC4-TSAR.1392) and stable (EXOC4-TSAR.2949) zebrafish showing the expression pattern of the reporter gene eGFP driven by the two accelerated elements in the *EXOC4* locus. We show the best representative image for each stable line and three representative EGFP expressing fishes in the transient analysis (C) Expression analysis of *EXOC4* in the inner ear. On the top left, a schematic of the inner ear showing the relative expression *EXOC4* in different regions of the mouse cochlea at E16.5 [5]. On the right, the graph shows the *EXOC4* expression level in the different cell types of the adult Organ of Corti in Deiters' cells, pillar cells, inner hair cells and outer hair cells (Liu et al. 2018). Below, the graphs show expression data for scRNA-seq on FACS sorted cells from the neuromast of 5dpf zebrafish embryos. The expression of exoc4 positive cells is depicted on the left diagram and the identified neuromast cell types are depicted on the right diagram [4]. Expression diagrams were obtained from the gEAR portal (www.umgear.org) [6] scRNAseq plots were generated from (https://piotrowskilab.shinyapps.io/neuromast_homeostasis_scrnaseq_2018).

Supplementary Figure S6. Enhancer assays in stable transgenic zebrafish for the SMOC1-TSAR3685 noncoding element.
(A) Panel showing the genome locus of the gene *SMOC1* in the human genome and the location of the TSAR elements in the UCSC Genome Browser. In the lower part of the panel GeneHancer and Conservation Tracks are displayed. The region containing SMOC1-TSAR3685 shows regulatory marks and evidence of interaction with the promoter of SMOC1 (B) Fluorescent microphotographs of six whole mount stable transgenic zebrafish lines showing the expression pattern of the reporter gene eGFP driven by the SMOC1-TSAR3685 element. We show the best representative image for each line. (C) In situ hybridization studies at 24, 48 and 72 hpf in zebrafish performed by [7]. In the left image, expression is observed in the brain and eye. In the central image, expression is observed in the eye. In the left image, expression is observed in the eye and pharyngeal arches. No strong expression is observed in the otic vesicle.
(D) Expression analysis of SMOC1 in the mouse inner ear. On the left, a schematic of the inner ear showing the relative expression SMOC1 in different regions of the mouse cochlea at embryonic day (E) E16.5 [5]. On the right, the

graph shows the SMOC1 expression level in the different cell types of the adult Organ of Corti in Deiters' cells, pillar cells, inner hair cells and outer hair cells [8]. Expression diagrams were obtained from the gEAR portal (www.umgear.org). Anatomic references: hb: hindbrain; mb: midbrain; hb: hindbrain; sc: spinal cord; oc: otic capsule; h: heart; ba: branchial arches; op: olfactory pits.

Supplementary Figure S7. Enhancer assays in stable transgenic zebrafish for the MIPOL1-TSAR.2840 noncoding element.
(A) Panel showing the genome locus of the gene *MIPOL1* in the human genome and the location of the TSAR elements. In addition, the figure shows supported interactions between the noncoding elements and the *MIPOL1* promoter predicted by GeneHancer track and conservation data in the UCSC Genome Browser. Some regulatory elements in the *MIPOL1* locus show interaction with the *MIPOL1* promoter. (B) Fluorescent microphotographs of four whole mount stable transgenic zebrafish lines showing the expression pattern of the reporter gene eGFP driven by the MIPOL1-TSAR.2840 element. We show the best representative image for each line. (C) On the right, the graph shows the *MIPOL1* expression level in the different cell types of the adult Organ of Corti in Deiters' cells, pillar cells, inner hair cells and outer hair cells [8]. On the central panel the graphs show expression data for scRNA-seq on FACS sorted cells from the neuromast of 5dpf zebrafish embryos. The expression of mipol1 positive cells is depicted on the central diagram and the identified neuromast cell types are depicted on the right diagram [4]. mipol1 is expressed in several neuromast cell types, mostly mature and young hair cells. Expression diagrams were obtained from the gEAR portal (www.umgear.org) and scRNAseq plots were generated from (https://piotrowskilab.shinyapps.io/neuromast_homeostasis_scrnaseq_2018). . Anatomic references: hb: hindbrain; mb: midbrain; hb: hindbrain; sc: spinal cord; oc: otic capsule; h: heart; ba: branchial arches; op: olfactory pits.

Supplementary Figure S8. Enhancer assays in stable transgenic zebrafish for the GATA2-TSAR.3936 noncoding element.
(A) Panel showing the genome locus of the gene *GATA2* in the human genome and the location of the TSAR elements, along with H3K27ac marks on 7 cell lines from ENCODE. (B) Fluorescent microphotographs of four whole mount stable transgenic zebrafish lines showing the expression pattern of the reporter gene eGFP driven by GATA2-TSAR.3936. We show the best representative image for each line. (C) In situ hybridization assays of gata2a performed at three stages of the zebrafish development (ZFIN, [9]). Expression is observed in the cranial region, hindbrain, tegmentum, otic vesicle, eye and pharyngeal arches. (D) Expression data for scRNA-seq on FACS sorted cells from the neuromast of 5dpf zebrafish embryos. The expression of gata2a and gata2b positive cells is depicted on the left and middle diagram respectively and the identified neuromast cell types are depicted on the right diagram [4]. Both gata2a and gata2b show expression on the central support cells of the neuromast. Expression diagrams were obtained from the gEAR portal (www.umgear.org) and scRNAseq plots

were generated from (https://piotrowskilab.shinyapps.io/neuromast_homeostasis_scrnaseq_2018). Anatomic references: hb: hindbrain; mb: midbrain; hb: hindbrain; sc: spinal cord; oc: otic capsule; h: heart; ba: branchial arches; op: olfactory pits.

Supplementary Figure S9. Comparative enhancer assays in transgenic zebrafish of the accelerated sequence JAZF1-TSAR.4204.
(A) Fluorescent microphotographs of whole mount stable transgenic zebrafish lines showing the expression pattern of the reporter gene eGFP driven by the human sequence of JAZF1-TSAR.4204 (Hs) (top, seven independent transgenic lines) and for the chicken ortholog JAZF1-TSAR.4204-Gg (below, three independent transgenic lines). White arrows in 72hpf lines indicate nascent neuromasts. We show the best representative image for each line (B) Expression data for scRNA-seq on FACS sorted cells from the neuromast of 5dpf zebrafish embryos. On the top left, the expression of jazf1a positive cells is depicted. On the bottom left, the expression of jazf1b positive cells is depicted. On the top right diagrams, the identified neuromast cell types are depicted [4]. jazf1a and jazf1b show no statistical significance differential expression for any particular cell type in the neuromast. (C) Relative expression of jazf1b in 5dpf zebrafish hair cells (IP) in comparison to whole larvae (IN) [10]. (D) Expression analysis of JAZF1 in the mouse inner ear. On the top left, a schematic of the inner ear showing the relative expression JAZF1 in different regions of the mouse cochlea at E16.5 [5]. (E) JAZF1 expression level in the different cell types of the adult Organ of Corti in Deiters' cells, pillar cells, inner hair cells and outer hair cells [8]. Expression diagrams were obtained from the gEAR portal (www.umgear.org). Anatomic references: hb: hindbrain; mb: midbrain; hb: hindbrain; sc: spinal cord; oc: otic capsule; h: heart; ba: branchial arches; op: olfactory pits.

Supplementary Figure S10. JAZF1-TSAR.4204-Hs directs the expression to neuromast in the developing zebrafish. Three independent stale transgenic lines carrying the JAZF1-TSAR.4204-Hs transgene directing the expression of the reporter gene eGFP to the neuromasts of the lateral line and also in the developing otic capsule (inner ear; IE). In the last column of pictures neuromasts are shown using the red fluorescent marker FM4-64 that specifically binds to hair cells. White arrowheads are used to indicate neuromast when they are not totally clear because of the high expression in other tissues (see line 1 on the top row and line 3 in the last row). In the first two rows neuromast of the head are shown using higher magnification whereas in the last row a detail of neuromast of the tail are shown in more detail. We show the best representative image for each line.

Supplementary Figure S11. Comparative analysis of genes under coding positive selection vs. non-coding acceleration in mammals. Analysis of overrepresentation of GO terms performed through gProfiler (https://biit.cs.ut.ee/gprofiler/gost) comparing genes showing positive selection in

their coding regions detected through PAML codeml in the mammalian lineage and genes carrying ncTSARs in their noncoding regions. In red, overrepresented terms for the genes containing ncTSARs are highlighted. In black, overrepresented terms for the genes containing positive selection in the mammalian lineage as detected with PAML codeml software. Only terms for the molecular function (MF) category are shown.

Supplementary Figure S12. Comparative analysis of genes with signatures of non-coding acceleration in mammals vs. non-coding acceleration in humans. Analysis of overrepresentation of GO terms performed through gProfiler (https://biit.cs.ut.ee/gprofiler/gost) comparing genes showing acceleration in their non-coding regions in the mammalian lineage (ncTSARs) and genes carrying human accelerated sequences (HARs) in their transcriptional units. In black, overrepresented terms for the genes containing ncTSARs are highlighted. In red, overrepresented terms for the genes containing HARs. Only terms for the molecular function (MF) category are shown.

Supplementary Figure S13. Analysis of overlap among different sets of genes and network association analyses.
(A) Intersection of the different sets of genes with evidence of selection (at either their coding or non-coding regions) in the mammalian and the human lineages. References: ncTSARs; non-coding acceleration at the mammalian lineage. Mammal cPS; positive selection at the coding regions in the mammalian lineage. HumancPS; positive selection at the coding regions in the human lineage. HARs; non-coding acceleration at the human lineage. (B) A network interaction map of the genes with signatures of coding acceleration at the mammalian lineage (cTSARs) obtained from the STRING database (https://string-db.org). Only a few of these cTSARs-containing genes show previous evidence of interaction. (C) A network interaction map of the genes associated with non-coding acceleration at the mammalian lineage (ncTSARs) and non-coding acceleration at the human lineage (HARs) from the STRING database (https://string-db.org). Several genes containing signatures of non-coding acceleration are shown to interact on the scheme. Color references: Edges represent protein associations, with either known or predicted interactions. Known interactions are represented by light-blue lines (curated databases) or purple lines (experimentally determined). Predicted interactions are represented by green (neighbors), red (fusions) and blue (co-occurrence) edges. Other interactions are represented by gold (text-mining), black (co-expression) and grey (protein-homology) lines.

References

1. Ganapathy A, Pandey N, Srisailapathy CRS, Jalvi R, Malhotra V, Venkatappa M, et al. Non-syndromic hearing impairment in India: high allelic heterogeneity among mutations in TMPRSS3, TMC1, USHIC, CDH23 and TMIE. PLoS One. 2014;9:e84773.

2. Michalski N, Goutman JD, Auclair SM, Boutet de Monvel J, Tertrais M, Emptoz A, et

al. Otoferlin acts as a Ca2+ sensor for vesicle fusion and vesicle pool replenishment at auditory hair cell ribbon synapses. Elife. 2017;6. doi:10.7554/eLife.31013.

3. Grillet N, Schwander M, Hildebrand MS, Sczaniecka A, Kolatkar A, Velasco J, et al. Mutations in LOXHD1, an evolutionarily conserved stereociliary protein, disrupt hair cell function in mice and cause progressive hearing loss in humans. Am J Hum Genet. 2009;85:328–37.

4. Lush ME, Diaz DC, Koenecke N, Baek S, Boldt H, St Peter MK, et al. scRNA-Seq reveals distinct stem cell populations that drive hair cell regeneration after loss of Fgf and Notch signaling. Elife. 2019;8. doi:10.7554/eLife.44431.

5. Rudnicki A, Isakov O, Ushakov K, Shivatzki S, Weiss I, Friedman LM, et al. Next-generation sequencing of small RNAs from inner ear sensory epithelium identifies microRNAs and defines regulatory pathways. BMC Genomics. 2014;15:484.

6. Orvis J, Gottfried B, Kancherla J, Adkins RS, Song Y, Dror AA, et al. gEAR: Gene Expression Analysis Resource portal for community-driven, multi-omic data exploration. Nat Methods. 2021;18:843–4.

7. Abouzeid H, Boisset G, Favez T, Youssef M, Marzouk I, Shakankiry N, et al. Mutations in the SPARC-related modular calcium-binding protein 1 gene, SMOC1, cause waardenburg anophthalmia syndrome. Am J Hum Genet. 2011;88:92–8.

8. Liu H, Chen L, Giffen KP, Stringham ST, Li Y, Judge PD, et al. Cell-Specific Transcriptome Analysis Shows That Adult Pillar and Deiters' Cells Express Genes Encoding Machinery for Specializations of Cochlear Hair Cells. Front Mol Neurosci. 2018;11:356.

9. Thisse B, Heyer V, Lux A, Alunni V, Degrave A, Seiliez I, et al. Spatial and temporal expression of the zebrafish genome by large-scale in situ hybridization screening. Methods Cell Biol. 2004;77:505–19.

10. Matern MS, Beirl A, Ogawa Y, Song Y, Paladugu N, Kindt KS, et al. Transcriptomic Profiling of Zebrafish Hair Cells Using RiboTag. Front Cell Dev Biol. 2018;6:47.
